# Supplementary material for: Association of Early Childhood Caries with Bitter Taste Receptors: A Meta-Analysis of Genome-Wide Association Studies and Transcriptome-Wide Association Study
Source: Genes (Basel). 2022 Dec 24;14(1):59. doi: 10.3390/genes14010059 (PMC9858612; doi:10.3390/genes14010059)
Supplement: Supplementary file 1 [file genes-14-00059-s001.zip › genes-2076649-supplementary.pdf]

Supplementary Methods

Participant Cohorts and Data Collection

COHRA1 participants come from underserved communities in West Virginia and Western Pennsylvania where more than half of children have early childhood caries at age 5[16]. COHRA1 participants were recruited through a household-based strategy, which necessitated having one biological parent-child pair in the house with the child between 1-18 years of age; subsequently all other members of the household were invited to participate. There were no exclusions in the study with respect to oral disease, but participants with neurological impairment, severe physical or mental disability and psychosis were excluded, as well as households where an original parent or offspring was immunocompromised (e.g., unstable diabetes) or had reduced ability to form blood clots. The standardized dental screening protocol was performed in an exam room by two staff members, either a dentist or dental hygienist and an assistant. The research was approved by the University of Pittsburgh Institutional Review Board (coordinating center approval # 0207073, Pennsylvania site approval # 0506048) and West Virginia University Institutional Review Board (approval # 15620B), and was in compliance with the Helsinki Declaration. Written informed consent of the parent and assent of the children was obtained[15]. Additional details about the procedures and caries status of this cohort have been previously described[4,58].

COHRA2 recruited healthy pregnant Caucasian women, and excluded those younger than 18 years of age, non-singleton pregnancies, and those with tuberculosis or immunocompromised. Women were withdrawn from the study if they delivered prematurely, before the 35th week of pregnancy, or if they or their baby developed a serious medical condition. If babies were born underweight, it was decided on a case-by-case basis with COHRA2 consulting pediatricians whether there were additional health problems that required removing the baby and mother from the study. Women were recruited from Pittsburgh hospitals (largely Magee Womens Hospital of UPMC) or from West Virginia hospitals. The exams were carried out by licensed dentists or dental hygienists with help from a research assistant. Dental examiners have been trained and calibrated on the tooth codes regularly from the beginning of the study, with substantial to excellent inter-rater reliability scores (Cohen's *kappa*, 82.1-92.5). Babies had research visits at birth, at age 2-3 months (pre-tooth visit), one month after the eruption of the first tooth (in Pittsburgh only), at 1 year of age, and every year thereafter. IRB approval was obtained from the University of Pittsburgh and West Virginia University. The study has an external review board, whose members contribute their expertise in pediatric dental research, genetics, and other realms[16]. The NIDCR provides oversight through a Clinical Study Oversight Committee (CSOC) and periodic site visits are performed by the Clinical Research Operations and Management Support (CROMS) system.

Among the children of the The Iowa Fluoride Study (IFS) there were no reports of malnourishment, and 4% of the children weighed less than 2500g and were designated low birth weight; none had very low (1000–1500g) or extremely low (< 1000g) birth weight [59]. The project was approved by the University of Iowa College of Dentistry Institutional Review Board.

The children enrolled in the Iowa Head Start (IHS) study lived in a community with optimally fluoridated water, and received two meals per day through Head Start. Informed consent was obtained from one or both parents, and the University of Iowa IRB approved the study.

ALSPAC, also known as "Children of the 90s," is a longitudinal birth cohort investigating a range of health, social, and developmental outcomes across the life course, and across generations. Demographically, the enrolled participants were more likely to have higher educational attainment at age 16 years, and less likely to be eligible for free school meals relative to a national sample[23]. Caries data were sourced from parent- or child-completed questionnaires between 38-91 months, and via dental exams for a random 10% of the cohort. Childrens' dental caries data were collected through questionnaires

administered to the parents when the children were 38, 54, and 65 months old, and to children when the children were 91 months old. At 31, 43, and 61 months of age, a random 10% of the childrens' cohort was chosen to participate in dental exams to collect dmft scores (decayed, missing, or filled primary teeth)[60]. Ethical approval for the study was obtained from the ALSPAC Ethics and Law Committee and the Local Research Ethics Committees (#B3063).

Genetic Data

DNA samples (buccal swab, blood, mouthwash, saliva) were isolated for participants in COHRA1, IFS, and IHS and genotyped for 580,000 genetic variants using the Illumina Human610-Quad Beadchip (Illumina, Inc., San Diego, CA). Genetic data were cleaned as previously described[8] and imputed to the 1000 Genomes Project (phase 1) reference panel[61] using SHAPEIT[62] and IMPUTE2[63]. 16.2 million SNPs were available for analysis. In individuals, imputed genotypes were filtered out if their probability was < 0.9, and SNPs with INFO score < 0.5 and SNPs that showed deviation from Hardy-Weinberg Equilibrium (HWE) with p-values less than 10^{-4} were removed.

COHRA2 DNA samples (saliva) were genotyped for 1.7M variants using the Infinium Multi-Ethnic Global-8 v1.0 array. SNPs were imputed by the Center for Inherited Disease Research (CIDR) and the University of Michigan Imputation Server[64] to the Haplotype Reference Consortium (HRC) reference panel [65] to yield 40.4M SNPs. Samples with greater than 5% missingness, and SNPs with Rsq < 0.5 and SNPs with HWE p-values less than 10^{-4} were removed.

Participants in ALSPAC were genotyped from blood or buccal swab samples for 550,000 SNPs on the Illumina HumanHap550 quad chip. ALSPAC data were cleaned and quality controlled as described previously[5]. Briefly, SNPs were filtered out with missingness more than 5%, HWE p-values less than $10e^{-5}$, and MAF <1%. Samples with greater than 5% missingness and indeterminate X chromosome heterozygosity were removed, and genomes were phased using ShapeIT (v2.r644) and imputed to HRC v1.1 using IMPUTE v2.2.2. All cohorts' SNPs were filtered for >5% minor allele frequency (MAF).

Functional Annotation of TWAS-Identified Transcripts

To gain more insight into the potential caries-related mechanisms, we investigated biological, functional and gene expression-related information of the genes corresponding to TWAS-identified transcripts using the GENE2FUNC process of FUMA[66].

Supplementary Results

Notable positional candidate genes within +/-500kb of lead suggestive SNPs in the meta-analysis of ECC.

Notable positional candidate genes include *CHST9*, which encodes carbohydrate sulfotransferase 9, and is the gene nearest rs8091366 ($p = 7.60\times10^{-6}$); it has previously been suggestively associated with caries of permanent dentition [13]. *SHISA9*, the closest gene to rs9889096 ($p = 5.59\times10^{-6}$), which encodes Protein Sihsa-9, is a regulator of synaptic plasticity. *SHISA9* was previously identified as a candidate gene for periodontitis due to the association of an upstream SNP (rs729876) with the trait [67]. *CASP7* (rs9889096; $p = 5.59\times10^{-6}$) encodes an apoptosis-related cysteine peptidase, which is involved in the mineralization of dental tissues; mouse knockouts of the gene cause a thinner layer of hard tissue in the adult mouse incisor[68]. *RPL21* (rs563135; $p = 5.86\times10^{-6}$) encodes 60S Ribosomal Protein L1, which participates in the development of tooth germ and synthesis of enamel components in mice [69], however in humans *RPL21* variants cause the autosomal dominant hair growth disorder, hypotrichosis-12, with phenotypically unaffected teeth[70]. NPVF (rs1044956; $p = 5.35E^{-6}$) encodes a neuronal propeptide that is expressed in brain regions that regulate affective aspects of pain, and is involved in the pain response [71]. *ST3GAL1* (rs76823412; $p = 1.54E^{-6}$) encodes a sialyltransferase that modifies glycans and is found in high quantities in saliva and oral mucosa. O-glycans of mucins are known to interact with microbes[72].

Gene set enrichment analysis

Enrichment analysis was performed against 18,777 genes. Input genes were (1) the four genes significantly associated (*CDH17, TAS2R43, SMIM10L1, TAS2R14*) and (2) the five genes suggestively associated (*CDH17, TAS2R43, SMIM10L1, TAS2R14, TAS2R31*) in the TWAS. No GO terms, phenotypes nor Ensembl genes were significantly associated with the set of significant nor the combined set of significant and suggestive genes identified by TWAS.

Supplementary Discussion

In addition to the protein-coding genes identified in the TWAS, two of the genes encode lncRNAs (*LINC02905, NRAD1)*, which, as a class, show functional regulatory versatility and potential to serve as disease biomarkers [73]. Both of these genes were not significant in individual tissue TWAS results, and so a conservative interpretation might be that they represent false positives due to LD misspecification, although it is also possible that these are modestly significant across multiple tissues and add up to a significant association in the meta-analysis.

Although no loci in the five GWASs of ECC nor the meta-analysis reached genome-wide significance for association, several loci showed some evidence for association, surpassing the suggestive significance threshold. As with previous GWASs of caries, the individual GWASs of ECC and the ECC meta-analysis identified genes with some evidence for association whose function is related to the multifactorial nature of the condition. Gene functions related to tooth morphology, immune response to bacteria, nociception, periodontal disease, and others were represented, and are detailed in **Table S3**.

Several loci associated with ECC were previously associated with caries in primary or permanent dentition (though they were not formally replicated in this study). This is in agreement with literature that suggests that genes affecting susceptibility to caries in primary dentition partly differ from those affecting susceptibility to permanent tooth caries[13], and suggests that genetic susceptibility to ECC may overlap with genetic susceptibility to caries across the life course. In the meta-analysis of ECC the SNP rs8091366 was nearest the gene *CHST9,* a positional candidate gene suggestively associated with caries of permanent dentition (rs9961915; r^2 with ECC lead SNP is 0.12,) [13]. In the COHRA1 GWAS, the locus harboring the SNP rs9301298 was previously suggestively associated with caries of primary dentition as tagged by rs17485138 [74], however rs17485138 is in low LD with the lead SNP in this study ($r^2 < 0.01$). Also in COHRA1, the rs35493737 locus was previously associated with primary tooth caries (dft score ≥ 1) in a partially overlapping cohort of children aged 3-12 years in COHRA1, IFS and IHS (lead SNP rs9907940); the lead SNP in Shaffer et al. is in low LD with the lead SNP at this locus ($r^2 < 0.2$) [8]. In the COHRA2 GWAS, the SNP rs11824206 is near *FBX03*, a gene associated with 5 GO terms derived from primary tooth caries GWAS data of COHRA1, IFS and IHS children aged 3-12 years [75]. The locus tagged by rs17227267 in COHRA2 was previously suggestively associated with caries (dft score and dfs score) in African American children (lead SNP rs2012033; in low LD with lead SNP at this locus, $r^2 = 0.012$) [12]. A cluster of beta-defensin anti-microbial proteins was near a signal for ECC at suggestive significance in the COHRA2 GWAS (rs2645455; *DEFB130, DEFB134, DEFB 135, DEFB136*). DEFB1-3 have previously been associated with caries or early childhood caries[12,76–78]. Interestingly, bitter taste receptors in sinonasal chemosensory cells are known to stimulate a rapid release of beta-defensins, in contrast to the slow and sustained beta-defensin upregulation due to TLR activation[44]. In the IHS cohort GWAS, the SNP rs11595665 is nearest the gene *PARD3*, which previously contributed to association of caries with the GO term "axonogenesis" based on SNPs derived from primary tooth caries GWAS data of COHRA1, IFS and IHS children aged 3-12 years [75], although the children in the IHS cohort in the previous study in part overlapped the children in our IHS GWAS.

Tables

Table S1. Statistical methods for GWAS analyses of ECC.

Cohort	Statistical Model	Covariates	Program	MAF cut-off	SNPs Tested
COHRA1	Mixed linear models	Age, sex, study site †	EMMAX	5%	5.9M
COHRA2	Mixed linear models	Age, sex, study site †	EMMAX	5%	4.4M
Iowa Fluoride Study (IFS)	Logistic regression assuming additive model	Age, sex, 1 principal component of ancestry	PLINK v1.9	5%	5.2M
Iowa Head Start (IHS)	Logistic regression assuming additive model	Age, sex, 1 principal component of ancestry	PLINK v1.9	5%	5.2M
ALSPAC	Mixed linear models	Age, sex †	BOLT-LMM for chr 1-22 and fastGWA in GCTA for the X chromosomes; X chromosomes were meta-analyzed in PLINK v1.9	5%	5.4M

† mixed linear models adjust for relatedness and ancestry. MAF minor allele frequency; SNP single nucleotide polymorphism

Table S2. Annotations for genes near SNPs suggestively associated (*p*-value < 1×10^{-5}) with ECC in the meta-analysis. Genes within a +/-500kb window of lead SNPs are annotated for potential relationship with ECC.

SNP	Chr:Pos	Select Gene(s)‡	Annotation
rs76823412	8:134690570	*ST3GAL1**	*ST3GAL1* encodes a sialyltransferase that modifies glycans and is expressed in the submandibular gland; O-glycans of mucins are known to interact with microbes[72]<br>Abnormal expression of *NDRG1* (N-myc downstream-regulate gene) is associated with metastasis in oral cancer [79]
rs74470773	3:33958083	*PDCD6IP**	N/A
rs1044956	7:24854765	*OSBPL3*, NPVF*	NPVF is involved in pain response; particularly expressed in brain regions regulating affective aspects of pain [71]
rs9889096	16:13575143	*SHISA9**	*SHISA9* is a candidate risk gene for periodontitis [67]
rs563135	10:115067899	*TCF7L2*, CASP7*	Variants in *TCF7L2* are associated with increased risk of type 2 diabetes [80]<br>CASP7 is involved in the development and differentiation of dental tissues [68]<br>Plekhs1 is a candidate mediator of onset of type 2 diabetes in obese rats [81]
rs7325099	13:28104496	*LNX2*, POLR1D, RPL21*	*POLR1D* variants cause Treacher Collins syndrome, a disorder of craniofacial development; features include mandibular hypoplasia and cleft palate [82]<br>Based on gene expression dynamics, Rpl21 may participate in development of tooth germ and synthesis of enamel components in mice [69]. Variants in the gene cause hypotrichosis-12, a disorder of hair growth [70].

rs8091366	18:24715618	*CHST9**, *KCTD1*	*CHST9* is a positional candidate gene suggestively associated with caries of permanent dentition (rs8094101, rs9961915) [13]. Variants in *KCTD1* cause Scalp-Ear-Nipple syndrome, which can less frequently cause dental anomalies [83]

Chr:Pos; chromosome and basepair position in GRCh37. ‡ Select genes within 500 kb of lead SNP. * Gene nearest associated SNP.

Table S3. Positional candidate gene annotations for suggestively significant SNPs in the five GWASs of ECC. Genes with a potential biological relationship with early childhood caries within a +/- 500 kb window of the lead SNP are annotated. Suggestive association threshold is *p*-value < 1×10^{-5}. The five cohorts in which GWASs were performed are demarcated in grey.

SNP	Chr:Pos	Select Gene(s) †	Annotation ‡
**COHRA1**			
rs28888981	8:57547011	*LINC00968**, *PENK*, *PLAG1*	LINC00968 promotes osteogenic differentiation of dental pulp stem cells; overexpression promotes mineralized bone matrix and other factors[84]<br>*PENK* encodes preproenkephalin, which is involved in central modulation of dental pain [85]<br>Aberrant *PLAG1* expression is found in almost half of salivary gland tumors [86]
rs9301298	13:109088897	*TNFSF13B**	*TNFSF13B* encodes a cytokine that is expressed at lower levels in individuals with Down syndrome who have periodontitis vs those who do not; it is also impaired in other conditions impacting the immune system[87]<br>This locus was suggestively associated with caries of permanent dentition (rs17485138) in COHRA1 [74], however that SNP is in low LD with the lead SNP in this study (r^2<0.01).
rs6557618	8:23001125	*TNFRSF10D**, *TNFRSF10C*, *R3HCC1*	*TNFRSF10C* was a moderately ranked gene in gene set enrichment analyses of GWASs of chronic periodontitis in European adults (ARIC study) [88].<br>*R3HCC1* was identified as a crosstalk gene within a module of periodontitis-associated genes [89]
rs9999579	4:57550252	*HOPX**	*hopx* expression is a stem cell marker of teeth and taste buds in cichlid fishes[90]
rs4289037	17:70617496	*LINC00511**, *SOX9*	*SOX9* is strongly expressed in dental pulp tissue, and reduced in inflamed pulp; it participates in regulation of

			extracellular matrix balance, the inflammatory process, and immune response [91]
			Rnf43 is a candidate gene for tooth development in mice [92]
			MPO encodes myeloperoxidase, which is involved in defense against periodontal bacteria; polymorphisms in *MPO* correlate with increased risk of aggressive periodontitis [34]
			LPO encodes lactoperoxidase, an enzyme involved in the nonspecific immune response in oral health maintenance [93]
rs35493737	17:56418136	*BZRAP1–AS1*, RNF43, MPO, LPO, OR4D2, OR4D1*	*OR4D1* and *OR4D2* encode olfactory receptors. The methylation patterns of *OR4D2* strongly correlate with daily intakes of total energy, carbohydrates, protein and fat [94]. Olfactory receptors are found throughout the body and respond to microbial metabolites[95].
			This locus is associated with primary tooth caries (dft score ≥ 1) in a partially overlapping cohort of children aged 3-12 years in COHRA1, IFS and IHS (lead SNP rs9907940); the lead SNP in Shaffer et al. is in low LD with the lead SNP at this locus ($r^2 < 0.2$) [8]
			This locus is associated with age at first tooth eruption (lead SNP rs412000, in low LD with lead SNP at this locus, r^2=0.15 in CEU in 1000G)[96]
**COHRA2**			
			FREM1 is part of the FREM2-FRAS1-FREM1 protein complex; variants in *FREM2* may disrupt the protein complex leading to dental and oral vestibule malformations [97]
rs56371878	9:14792016	*FREM1*, CER1, NFIB*	This locus is also associated with central upper lip height measures (lead SNP rs72713618, in low LD with lead SNP at this locus, r^2<0.01)[98]
			Variation in *CER1* is associated with bone mineral density [99]

			NFIB regulates embryonic development of submandibular salivary glands [100]
rs11824206	11:33933115	*LMO2**, *FBX03*, *CD59*, *CAPRIN*	*FBX03* was associated with 5 GO terms derived from primary tooth caries GWAS data of COHRA1, IFS and IHS children aged 3-12 years [75]<br><br>CD59 is a complement regulatory protein, whose gene expression is upregulated by periodontopathogen lipopolysaccharide and proinflammatory cytokines in oral epithelial cells [101]<br><br>CAPRIN is a stress granule-associated protein which possibly binds to PKR; PKR is the core protein of the dental pulp cell-derived powerful inducer of TNF-α (DPIT) [102]
rs12905748	15:58623465	*LIPC**, *ADAM*, *AQP9*, *ALDH1A2*	Variants in *LIPC* are associated with visceral adiposity indicators and triglyceride and fasting plasma glucose index-related parameters, mediated by serum triglyceride levels [103]. *LIPC* is also a candidate susceptibility gene for orofacial clefts and dental anomalies (tooth agenesis, supernumerary teeth, microdontia) [104]<br><br>Variants in *ADAM* cause Adams-Oliver syndrome 1, an autosomal dominant syndrome that includes cleft lip and cleft palate. *ADAM* is expressed by ameloblasts and facilitates enamel development [105]<br><br>AQP9 is upregulated in pulp tissue in, and considered a biomarker for irreversible pulpitis [106]<br><br>*Aldh1a2* is the key enzyme in retinoic acid production, and is expressed by dental follicle cells; retinoic acid controls dental development and self-renewal [107]
rs2645455	8:11619635	*C8orf49**, *NEIL2*, *CTSB*, *DEFB135*, *DEFB136*, *DEFB134*, *DEFB130*	*C8orf49* (aka *LINC02905*) is a gene identified in the TWAS of the ECC GWAS meta-analysis in this study.<br><br>*NEIL2* encodes a glycosylase that suppresses *Fusobacterium nucleatum* (a periodontal pathogen) infection-induced inflammation and DNA damage [108]

			CTSB encodes the most widely expressed cathepsin (a lysosomal protease); it is found at high levels in macrophages and gingival crevicular fluid; it plays a role in resorption lacunae formation in deciduous teeth, in dentin-pulp complex, and in ameloblasts [109]
			The β-defensin family (DEFB-) encode antimicrobial proteins present in saliva and other representatives such as *DEFB1* are associated with caries and oral carcinogenesis, and DEFB2 and DEFB3 levels with early childhood caries [12,76–78]
rs72800208	10:67045132	*LOC101928913**	N/A
rs1505638	9:81196896	*PSAT1**	*PSAT1* encodes a serine metabolism-related enzyme; the expression of *PSAT1* is decreased in adult dental pulp stem cells relative to those of the young; serine metabolism participates in stem cell aging [110]
rs78802022	20:821750	*FAM110A*, SRXN1, RSPO4*	*SRXN1* is highly down-regulated in response to human dental pulp stem cells treated with a low-level toxic conenctration of Triethylene Glycol Dimethacrylate (TEGDMA), a dentin resin restoration treatment [111]<br>*Rspo4* is differentially expressed in the developing mouse molar tooth (specifically, the dental papilla) relative to 16 control tissues [112]
rs17227267	19:34069736	*CHST8**	*CHST8* is a candidate gene for hypodontia [113]<br>This locus is suggestively associated with caries (dft score and dfs score) in African American children (lead SNP rs2012033; in low LD with lead SNP at this locus, r^2=0.012) [12]
rs3014919	13:46140665	*ERICH6B*, SIAH3*	A SNP at this locus is associated with obesity and carbohydrate preference. The SNP rs368794 is in moderate LD with the lead SNP at this locus (r^2<0.01)[114]<br>*SIAH3* is one of the most decreased genes in expression in healing gingiva, relative to normal gingiva [115]
rs35261848	4:95632043	*PDLIM5*, BMPR1B*	*BMPR1B* encodes a bone morphogenic protein; it is expressed in the developing dental epithelium of upper and lower

			incisor germs, as well as epithelium and mesenchyme. It has a high level of expression in the future inner enamel organ of molars [116].
**IFS**			
rs72625020	3:185596778	*TRA2B*, ETV5, IGF2BP2*	*Etv5* is expressed in the dental epithelium during tooth development [117]<br><br>*IGF2BP2* is targeted by let-7a to regulate human dental pulp cells' proliferation [118]
rs9677362	2:1625705	*PXDN**	This locus is associated with erosive tooth wear (lead SNP rs11681214; in low LD (r^2<0.01) with lead SNP at this locus) [119]
rs12581319	12:54333682	*HOXC13*, HOXC12, ZNF385A, ITGA5, NPFF*	*Hoxc13* encodes a transcription factor active in the dental epithelium of the developing tooth [120]<br><br>Hoxc12 is also expressed in the dental epithelium of the developing tooth [117]<br><br>*ZNF385A* is downregulated in dental pulp tissue, compared with periodontal ligament tissue in humans [121]<br><br>*ITGA5* expression influences proliferation, migration capacity, and odontogenic differentiation in human dental pulp stem cells [122]<br><br>NPFF is a neuropeptide that may be involved in nociception and may contribute to enhanced analgesic potency of morphine in peripheral inflammation [123]
rs78413413	18:64800322	*MIR5011*, DSEL*	*DSEL* is up-regulated (3.96x) in the dental follicle compared with periodontal ligament tissue [124]
rs13030273	2:167246067	*SCN7A*, SCN9A*	*SCN7A* is differentially expressed in dental pulp than in gingiva [125]<br><br>SCN9A plays an important role in pain and nociception; variants in SCN9A are associated with neuropathic pain [126]. 8

			SNPs tested in this gene did not show association with dental pain sensitivity in autistic children [127]
rs113573396	23:53816598	*HUWE1*, PHF8,*	HUWE1 variants are associated with a rare X-linked syndrome comprising intellectual disability, dysmorphic features, and oligodontia [128]<br>Variants in PHF8 are associated with Siderius X-linked mental retardation syndrome, which includes intellectual disability, dysmorphic facial features, and cleft lip/palate [129]
**IHS**			
rs9609768	22:33758736	*LARGE*(aka *LARGE1*)*, *TIMP3*	*TIMP* gene family members regulate matrix metalloproteinases, which play a central role in the control or progression of carious decay [130]. *TIMP3* is expressed in mesenchymal tissues and in dental epithelial cells throughout murine tooth development [131]
rs4331007	10:132690286	*MIR378C**	N/A
rs11595665	10:34890830	*PARD3**	*PARD3* contributed to association of caries with the GO term "axonogenesis" based on SNPs derived from primary tooth caries GWAS data of COHRA1, IFS and IHS children aged 3-12 years [75]
rs1455320	23:6184334	*NLGN4X**	N/A
rs1594693	19:32617999	*LINC01782**	N/A
rs2207244	14:26558936	*NOVA1**	N/A
**ALSPAC**			
rs3111217	1:218725751	*TGFB2**	TGF-beta 2 stimulates the synthesis and deposition of matrix in teeth in mice [132]. The TGF-beta family is known to be involved in tooth development and repair, and TGF-beta 2 has previously been proposed to play a role in caries [133]
rs255632	7:25094095	*CYCS*, NPVF*	NPVF is a neuropeptide with important roles in nociception, anxiety, learning and memory, and cardiovascular reflex. NPVF encodes two peptides that are highly similar to *NPFF* (a positional candidate gene on chr 12 near rs12581319 in GWAS in IFS in this study) [71]

rs56041987	17:74981998	*CDR2L*, ATP5PD, GRB2, OTOP2, GRIN2C, RAB37*	*ATP5PD* is a hub, interacting with 22 proteins in the protein-protein interaction network of the process of odontogenic differentiation in dental pulp stem cells [134]<br><br>*Grb2* expression increases in response to LPS-treated odontoblast-like dental pulp cells [135]<br><br>*Otop2* is transiently upregulated and then downregulated during ameloblast differentiation [136]<br><br>Human periodontal ligament cells constitutively express mRNA for *GRIN2C*, and mechanical stress up-regulates it [137]<br><br>*RAB37* is a target of a predicted eQTL (rs10456847) suggestively associated with chronic periodontitis in HCHS/SOL cohort adults [138]
rs7970173	12:105378181	*C12orf75*, APPL2*	*C12orf75* is up-regulated (4.14x) in the dental follicle compared with periodontal ligament tissue [124]<br><br>rs1196764 at *APPL2* is associated with tongue dorsum microbiota (*Prevotella jejuni, Oribacterium uSGB 3339 and Solobacterium uSGB 315*). This SNP is in low LD with the lead SNP at this locus (r^2=0.01). The 3 taxa correlate with high sugar/fat dietary frequency and decreased risk of dental calculus and gingival bleeding. APPL2 is involved in controlling glucose-stimulated insulin secretion, and is involved in negatively regulating inflammation [139]

† Select genes within 500 kb of lead SNP. ‡ Where noted, r^2 values are indicated for the CEU population of 1000 Genomes. * Gene nearest associated SNP.

Table S4. Gene annotations for transcripts associated with ECC as identified by TWAS. Genes encoding transcripts associated at significant (p-value < 3.09×10^{-6}) or suggestive (p-value < 1.0×10^{-4}) significance levels in S-MultiXcan analysis are shown. Genes are annotated for any relationship with early childhood caries.

Gene	Locus	Gene Start Chr:Pos*	Gene Annotation
LINC02905 (C8Orf49)‡	chr8p23.1	8:11618765	*LINC02905* is the nearest gene to a SNP that may interact with obesity in pancreatic cancer [105]; it is a putative risk gene for PCOS [106]
CDH17	chr8q22.1	8:95139394	*CDH17* encodes a peptide transporter in the small intestine [107]. Deletion of *CDH17* enhances intestinal permeability[108]
TAS2R43	chr12p13.2	12:11243886	Variants in *TAS2R43* correlate with liking coffee [109] TAS2Rs are important to the immune response; *TAS2R43* (along with *TAS2R31* and *TAS2R38*, discussed below) are expressed in polymorphonuclear neutrophils, the earliest immune cells recruited to the site of inflammation [57] Variants in other *TAS2R* family member was previously associated with dental caries (*TAS2R38*) [110]
SMIM10L1	chr12p13.2	12:11323780	N/A
TAS2R14	chr12p13.2	12:11090853	TAS2R14 detects quorum-sensing molecules from *S. mutans* and induces innate immune responses in gingival epithelial cells [111] TAS2R14 recognizes the most broad range of chemically diverse agonists, including caffeine, of all the bitter taste receptors; it is thought to play a role in innate immune responses [112]
***NRAD1 (LINC00284)*‡**	chr13q14.11	13:44596471	N/A
TAS2R31	chr12p13.2	12:11182986	SNPs in *TAS2R31* associated with whole-mouth quinine bitterness and self-reported liking for grapefruit juice [113]
LACC1‡	chr13q14.11	13:44453420	LACC1 plays a critical role in innate immunity in ER stress-dependent events in macrophages; *LACC1* variants are associated with inflammatory bowel disease, leprosy, Behcet disease, and juvenile arthritis. It is upregulated by bacterial LPS and other TLR ligands [114]
IGSF5‡	chr21q22.2	21:41117334	*EGSF5* encodes immunoglobulin superfamily, member 5, a tight junction adhesion protein. Overexpression of *EGSF5* is associated with worse prognosis in advanced colorectal adenomas [115]

* Gene start position in chromosome:basepair format, in GRCh37 coordinates. **Bolded** genes are associated with ECC at significant levels ($p < 3.09 \times 10^{-6}$), the rest at suggestive levels ($p < 1.00 \times 10^{-4}$) in the all-tissue TWAS. ‡ Genes that do not surpass suggestive significance in any individual gene-tissue analysis ($p < 1.0 \times 10^{-4}$); these genes may be identified in the all-tissue TWAS due to LD misspecification.

Table S5. Details of results of TWAS of ECC meta-analysis. Genes encoding transcripts associated at significant (p-value < $3.09{\times}10^{-6}$) or suggestive (*p*-value < $1.0{\times}10^{-4}$) significance levels in S-MultiXcan analysis are shown.

Gene name	P-value	N	N indep.	Best p-value	Best tissue	Mean Z	Z SD
C8orf49	1.82×10^{-8}	9	3	0.105	Heart_Atrial_Appendage	-0.35	1.33
CDH17	3.32×10^{-8}	23	5	9.40×10^{-10}	Nerve_Tibial	1.74	3.00
TAS2R43	8.78×10^{-8}	27	3	4.69×10^{-9}	Breast_Mammary_Tissue	1.80	1.89
SMIM10L1	1.70×10^{-7}	46	3	8.99×10^{-9}	Colon_Sigmoid	-0.35	1.62
TAS2R14	1.06×10^{-6}	48	4	8.99×10^{-9}	Brain_Caudate_basal_ganglia		1.27
LINC00284	1.69×10^{-6}	30	5	0.067	Testis	0.13	1.07
TAS2R31	1.04×10^{-5}	41	4	5.05×10^{-6}	Prostate	-0.25	1.49
LACC1	3.98×10^{-5}	48	4	0.001	Cells_Cultured_fibroblasts	0.10	1.16
IGSF5	7.22×10^{-5}	27	11	0.005	Lung	0.63	1.40

P-value; p-value of S-MultiXcan association. N; number of tissues available for gene. N indep; number of independent components of variation kept among the tissues' predictions. Best p-value; best p-value of single-tissue S-PrediXcan association. Best tissue; best single-tissue S-PrediXcan association. Mean Z; mean z-score among single-tissue S-PrediXcan associations. Z SD; standard deviation of the mean z-score among single-tissue S-PrediXcan associations.

Table S6. Colocalization analysis probabilities of ECC GWAS SNPs and GTEx eQTLs across 49 tissues. Posterior probabilities of colocalization of GWAS-identified signals and eQTLs spanning a 1 MB window around TWAS-identified genes for 214 gene-tissue pairs are shown. Some SNPs tested for colocalization reside within 1MB of multiple TWAS-identified genes, which is reflected in the Genes column. The posterior probabilities (PP) of five hypotheses (H) were tested: neither the GWAS nor any eQTLs have associations in the region (H0/null), only the GWAS (H1) or eQTL (H2) has genetic association in the region, both GWAS and eQTL are associated, but with different causal variants (H3), or both GWAS and eQTL are associated and share a single causal variant (H4). Evidence of SNP colocalization was defined by posterior probabilities of colocalization of (1) PP of H4 > 0.5, (2) PP of H3 < 0.5, and (3) PP of H0+H1+H2 <0.3. Results are sorted in descending order by PP.H4.

Genes	N SNPs	Tissue	PP.H0	PP.H1	PP.H2	PP.H3	PP.H4
C8Orf49	10	Heart_Left_Ventricle	$8.54{\rm x}10^{-14}$	$4.88{\rm x}10^{-18}$	$9.58{\rm x}10^{-1}$	$1.25{\rm x}10^{-5}$	$4.23{\rm x}10^{-2}$
IGSF5	3	Brain_Hippocampus	$3.86{\rm x}10^{-3}$	$1.30{\rm x}10^{-7}$	$9.74{\rm x}10^{-1}$	$1.09{\rm x}10^{-5}$	$2.20{\rm x}10^{-2}$
IGSF5	4	Adrenal_Gland	$5.85{\rm x}10^{-5}$	$1.91{\rm x}10^{-9}$	$9.88{\rm x}10^{-1}$	$2.02{\rm x}10^{-5}$	$1.22{\rm x}10^{-2}$
IGSF5	8	Pituitary	$2.50{\rm x}10^{-17}$	$1.23{\rm x}10^{-20}$	$9.95{\rm x}10^{-1}$	$4.86{\rm x}10^{-4}$	$4.52{\rm x}10^{-3}$
IGSF5	6	Vagina	$1.25{\rm x}10^{-9}$	$1.72{\rm x}10^{-14}$	$9.96{\rm x}10^{-1}$	$9.20{\rm x}10^{-6}$	$4.49{\rm x}10^{-3}$
IGSF5	5	Heart_Left_Ventricle	$1.47{\rm x}10^{-1}$	$1.74{\rm x}10^{-6}$	$8.49{\rm x}10^{-1}$	$5.62{\rm x}10^{-6}$	$4.41{\rm x}10^{-3}$
IGSF5	5	Brain_Spinal_cord_cervical_c-1	$3.70{\rm x}10^{-10}$	$5.28{\rm x}10^{-15}$	$9.96{\rm x}10^{-1}$	$1.02{\rm x}10^{-5}$	$3.96{\rm x}10^{-3}$
IGSF5	8	Ovary	$2.73{\rm x}10^{-6}$	$5.55{\rm x}10^{-11}$	$9.96{\rm x}10^{-1}$	$1.64{\rm x}10^{-5}$	$3.89{\rm x}10^{-3}$
TAS2R43, SMIM10L1, TAS2R14, TAS2R31	19	Pituitary	$5.11{\rm x}10^{-25}$	$1.17{\rm x}10^{-29}$	$9.97{\rm x}10^{-1}$	$1.96{\rm x}10^{-5}$	$3.21{\rm x}10^{-3}$
C8Orf49	16	Brain_Cortex	$8.59{\rm x}10^{-15}$	$2.07{\rm x}10^{-19}$	$9.97{\rm x}10^{-1}$	$2.14{\rm x}10^{-5}$	$2.68{\rm x}10^{-3}$
CDH17	8	Thyroid	$1.08{\rm x}10^{-14}$	$1.82{\rm x}10^{-19}$	$9.97{\rm x}10^{-1}$	$1.41{\rm x}10^{-5}$	$2.65{\rm x}10^{-3}$
IGSF5	4	Esophagus_Gastroesophageal_Junction	$5.58{\rm x}10^{-30}$	$3.37{\rm x}10^{-35}$	$9.97{\rm x}10^{-1}$	$3.41{\rm x}10^{-6}$	$2.61{\rm x}10^{-3}$
IGSF5	6	Lung	$5.22{\rm x}10^{-26}$	$3.81{\rm x}10^{-31}$	$9.97{\rm x}10^{-1}$	$4.70{\rm x}10^{-6}$	$2.58{\rm x}10^{-3}$
IGSF5	5	Artery_Aorta	$2.80{\rm x}10^{-21}$	$3.07{\rm x}10^{-24}$	$9.96{\rm x}10^{-1}$	$1.09{\rm x}10^{-3}$	$2.56{\rm x}10^{-3}$
IGSF5	7	Breast_Mammary_Tissue	$1.54{\rm x}10^{-40}$	$2.17{\rm x}10^{-45}$	$9.97{\rm x}10^{-1}$	$1.15{\rm x}10^{-5}$	$2.55{\rm x}10^{-3}$
IGSF5	5	Pancreas	$3.33{\rm x}10^{-4}$	$2.97{\rm x}10^{-9}$	$9.97{\rm x}10^{-1}$	$6.37{\rm x}10^{-6}$	$2.54{\rm x}10^{-3}$

IGSF5	6	Nerve_Tibial	1.02x10^{-61}	3.21x10^{-66}	9.97x10^{-1}	2.88x10^{-5}	2.52x10^{-3}
IGSF5	11	Cells_Cultured_fibroblasts	1.11x10^{-59}	2.15x10^{-64}	9.98x10^{-1}	1.68x10^{-5}	2.45x10^{-3}
C8Orf49	6	Liver	1.30x10^{-2}	1.37x10^{-7}	9.85x10^{-1}	7.97x10^{-6}	2.42x10^{-3}
IGSF5	4	Artery_Coronary	1.38x10^{-19}	1.63x10^{-24}	9.98x10^{-1}	9.46x10^{-6}	2.38x10^{-3}
IGSF5	9	Adipose_Subcutaneous	3.51x10^{-86}	1.32x10^{-90}	9.98x10^{-1}	3.51x10^{-5}	2.34x10^{-3}
IGSF5	6	Brain_Putamen_basal_ganglia	8.63x10^{-7}	1.21x10^{-11}	9.98x10^{-1}	1.17x10^{-5}	2.30x10^{-3}
IGSF5	6	Esophagus_Muscularis	1.70x10^{-41}	5.06x10^{-46}	9.98x10^{-1}	2.75x10^{-5}	2.30x10^{-3}
IGSF5	7	Prostate	2.17x10^{-8}	1.85x10^{-13}	9.98x10^{-1}	6.24x10^{-6}	2.25x10^{-3}
IGSF5	9	Brain_Cerebellar_Hemisphere	1.92x10^{-21}	2.70x10^{-26}	9.98x10^{-1}	1.18x10^{-5}	2.23x10^{-3}
IGSF5	5	Brain_Cerebellum	2.23x10^{-33}	2.31x10^{-38}	9.98x10^{-1}	8.14x10^{-6}	2.17x10^{-3}
IGSF5	7	Artery_Tibial	1.76x10^{-87}	1.86x10^{-92}	9.98x10^{-1}	8.38x10^{-6}	2.16x10^{-3}
IGSF5	7	Whole_Blood	3.04x10^{-37}	3.84x10^{-42}	9.98x10^{-1}	1.05x10^{-5}	2.15x10^{-3}
IGSF5	9	Thyroid	6.12x10^{-33}	2.34x10^{-37}	9.98x10^{-1}	3.60x10^{-5}	2.14x10^{-3}
IGSF5	9	Adipose_Visceral_Omentum	5.68x10^{-50}	1.96x10^{-54}	9.98x10^{-1}	3.23x10^{-5}	2.14x10^{-3}
C8Orf49	24	Testis	9.98x10^{-23}	3.69x10^{-27}	9.98x10^{-1}	3.48x10^{-5}	2.14x10^{-3}
CDH17	2	Heart_Left_Ventricle	5.42x10^{-2}	1.90x10^{-7}	9.44x10^{-1}	1.19x10^{-6}	2.13x10^{-3}
IGSF5	3	Minor_Salivary_Gland	8.27x10^{-2}	9.21x10^{-7}	9.15x10^{-1}	8.21x10^{-6}	1.98x10^{-3}
C8Orf49	14	Esophagus_Muscularis	4.01x10^{-27}	1.27x10^{-31}	9.98x10^{-1}	2.97x10^{-5}	1.92x10^{-3}
CDH17	6	Esophagus_Mucosa	2.68x10^{-3}	5.09x10^{-7}	9.95x10^{-1}	1.87x10^{-4}	1.87x10^{-3}
C8Orf49	11	Brain_Amygdala	5.38x10^{-2}	7.51x10^{-7}	9.44x10^{-1}	1.13x10^{-5}	1.85x10^{-3}
IGSF5	5	Brain_Caudate_basal_ganglia	2.83x10^{-2}	3.31x10^{-7}	9.70x10^{-1}	9.53x10^{-6}	1.82x10^{-3}
TAS2R43, SMIM10L1, TAS2R14, TAS2R31	14	Brain_Anterior_cingulate_cortex_BA24	1.77x10^{-9}	4.22x10^{-14}	9.98x10^{-1}	2.21x10^{-5}	1.74x10^{-3}
CDH17	5	Artery_Coronary	2.45x10^{-2}	2.47x10^{-6}	9.74x10^{-1}	9.63x10^{-5}	1.69x10^{-3}
TAS2R43, SMIM10L1, TAS2R14, TAS2R31	16	Brain_Frontal_Cortex_BA9	4.26x10^{-14}	1.75x10^{-18}	9.98x10^{-1}	3.94x10^{-5}	1.66x10^{-3}
C8Orf49	10	Lung	3.16x10^{-22}	5.65x10^{-27}	9.98x10^{-1}	1.62x10^{-5}	1.62x10^{-3}
IGSF5	5	Brain_Hypothalamus	1.42x10^{-1}	9.53x10^{-7}	8.57x10^{-1}	4.14x10^{-6}	1.62x10^{-3}
C8Orf49	9	Colon_Transverse	4.61x10^{-9}	9.90x10^{-14}	9.98x10^{-1}	1.98x10^{-5}	1.62x10^{-3}
C8Orf49	13	Spleen	4.75x10^{-12}	8.39x10^{-17}	9.98x10^{-1}	1.60x10^{-5}	1.62x10^{-3}
NRAD1, LACC1	5	Heart_Atrial_Appendage	1.92x10^{-58}	1.26x10^{-63}	9.98x10^{-1}	5.03x10^{-6}	1.53x10^{-3}
TAS2R43, SMIM10L1, TAS2R14, TAS2R31	14	Kidney_Cortex	6.30x10^{-3}	1.48x10^{-7}	9.92x10^{-1}	2.17x10^{-5}	1.52x10^{-3}
NRAD1, LACC1	8	Brain_Spinal_cord_cervical_c-1	2.81x10^{-13}	3.04x10^{-18}	9.98x10^{-1}	9.29x10^{-6}	1.52x10^{-3}
C8Orf49	11	Brain_Cerebellum	1.64x10^{-29}	3.33x10^{-34}	9.98x10^{-1}	1.87x10^{-5}	1.52x10^{-3}
NRAD1, LACC1	8	Adipose_Subcutaneous	6.66x10^{-69}	2.82x10^{-73}	9.98x10^{-1}	4.08x10^{-5}	1.51x10^{-3}
NRAD1, LACC1	5	Ovary	9.27x10^{-9}	6.47x10^{-14}	9.98x10^{-1}	5.46x10^{-6}	1.51x10^{-3}
NRAD1, LACC1	9	Lung	7.29x10^{-123}	1.67x10^{-127}	9.99x10^{-1}	2.15x10^{-5}	1.46x10^{-3}
NRAD1, LACC1	6	Muscle_Skeletal	6.46x10^{-128}	3.77x10^{-133}	9.99x10^{-1}	4.36x10^{-6}	1.46x10^{-3}

NRAD1, LACC1	5	Artery_Aorta	$4.98x10^{-71}$	$3.03x10^{-76}$	$9.99x10^{-1}$	$4.62x10^{-6}$	$1.45x10^{-3}$
NRAD1, LACC1	8	Skin_Sun_Exposed_Lower_leg	$5.18x10^{-123}$	$6.11x10^{-128}$	$9.99x10^{-1}$	$1.03x10^{-5}$	$1.45x10^{-3}$
NRAD1, LACC1	9	Artery_Coronary	$1.29x10^{-41}$	$1.55x10^{-46}$	$9.99x10^{-1}$	$1.05x10^{-5}$	$1.44x10^{-3}$
NRAD1, LACC1	9	Colon_Transverse	$1.98x10^{-56}$	$2.66x10^{-61}$	$9.99x10^{-1}$	$1.20x10^{-5}$	$1.44x10^{-3}$
NRAD1, LACC1	9	Cells_Cultured_fibroblasts	$2.50x10^{-107}$	$1.15x10^{-111}$	$9.99x10^{-1}$	$4.43x10^{-5}$	$1.44x10^{-3}$
NRAD1, LACC1	6	Nerve_Tibial	$2.02x10^{-127}$	$1.61x10^{-132}$	$9.99x10^{-1}$	$6.51x10^{-6}$	$1.44x10^{-3}$
NRAD1, LACC1	2	Pancreas	$3.78x10^{-52}$	$2.60x10^{-57}$	$9.99x10^{-1}$	$5.43x10^{-6}$	$1.43x10^{-3}$
C8Orf49	10	Breast_Mammary_Tissue	$2.25x10^{-8}$	$2.97x10^{-13}$	$9.99x10^{-1}$	$1.18x10^{-5}$	$1.43x10^{-3}$
NRAD1, LACC1	8	Brain_Amygdala	$3.61x10^{-24}$	$6.62x10^{-29}$	$9.99x10^{-1}$	$1.69x10^{-5}$	$1.43x10^{-3}$
NRAD1, LACC1	6	Esophagus_Mucosa	$2.33x10^{-116}$	$3.77x10^{-121}$	$9.99x10^{-1}$	$1.47x10^{-5}$	$1.43x10^{-3}$
NRAD1, LACC1	7	Breast_Mammary_Tissue	$2.69x10^{-60}$	$2.18x10^{-65}$	$9.99x10^{-1}$	$6.65x10^{-6}$	$1.43x10^{-3}$
NRAD1, LACC1	4	Esophagus_Muscularis	$1.44x10^{-84}$	$1.16x10^{-89}$	$9.99x10^{-1}$	$6.60x10^{-6}$	$1.42x10^{-3}$
NRAD1, LACC1	3	Heart_Left_Ventricle	$2.64x10^{-59}$	$3.62x10^{-64}$	$9.99x10^{-1}$	$1.23x10^{-5}$	$1.42x10^{-3}$
CDH17	2	Nerve_Tibial	$2.06x10^{-3}$	$6.36x10^{-9}$	$9.97x10^{-1}$	$1.67x10^{-6}$	$1.41x10^{-3}$
C8Orf49	7	Adrenal_Gland	$1.35x10^{-4}$	$1.27x10^{-9}$	$9.98x10^{-1}$	$8.01x10^{-6}$	$1.40x10^{-3}$
CDH17	6	Adipose_Subcutaneous	$1.55x10^{-7}$	$9.06x10^{-13}$	$9.99x10^{-1}$	$4.47x10^{-6}$	$1.37x10^{-3}$
C8Orf49	11	Esophagus_Gastroesophageal_Junction	$9.65x10^{-16}$	$1.19x10^{-20}$	$9.99x10^{-1}$	$1.09x10^{-5}$	$1.37x10^{-3}$
TAS2R43, SMIM10L1, TAS2R14, TAS2R31	24	Skin_Sun_Exposed_Lower_leg	$8.05x10^{-87}$	$2.96x10^{-91}$	$9.99x10^{-1}$	$3.54x10^{-5}$	$1.34x10^{-3}$
C8Orf49	10	Brain_Frontal_Cortex_BA9	$1.18x10^{-3}$	$1.46x10^{-8}$	$9.97x10^{-1}$	$1.10x10^{-5}$	$1.32x10^{-3}$
C8Orf49	11	Artery_Coronary	$3.58x10^{-4}$	$4.65x10^{-9}$	$9.98x10^{-1}$	$1.17x10^{-5}$	$1.29x10^{-3}$
NRAD1, LACC1	9	Vagina	$2.74x10^{-18}$	$1.10x10^{-22}$	$9.99x10^{-1}$	$3.87x10^{-5}$	$1.26x10^{-3}$
NRAD1, LACC1	9	Uterus	$2.63x10^{-10}$	$2.68x10^{-15}$	$9.99x10^{-1}$	$8.92x10^{-6}$	$1.26x10^{-3}$
C8Orf49	11	Muscle_Skeletal	$4.36x10^{-13}$	$4.81x10^{-18}$	$9.99x10^{-1}$	$9.79x10^{-6}$	$1.23x10^{-3}$
C8Orf49	10	Brain_Cerebellar_Hemisphere	$5.76x10^{-24}$	$1.74x10^{-28}$	$9.99x10^{-1}$	$2.89x10^{-5}$	$1.23x10^{-3}$
C8Orf49	12	Cells_EBV-transformed_lymphocytes	$1.15x10^{-5}$	$2.77x10^{-10}$	$9.99x10^{-1}$	$2.28x10^{-5}$	$1.23x10^{-3}$
C8Orf49	12	Brain_Anterior_cingulate_cortex_BA24	$6.95x10^{-12}$	$1.22x10^{-16}$	$9.99x10^{-1}$	$1.63x10^{-5}$	$1.23x10^{-3}$
NRAD1, LACC1	3	Brain_Putamen_basal_ganglia	$1.48x10^{-40}$	$5.80x10^{-46}$	$9.99x10^{-1}$	$2.68x10^{-6}$	$1.23x10^{-3}$
NRAD1, LACC1	4	Brain_Frontal_Cortex_BA9	$1.26x10^{-29}$	$5.50x10^{-35}$	$9.99x10^{-1}$	$3.12x10^{-6}$	$1.23x10^{-3}$
NRAD1, LACC1	5	Brain_Anterior_cingulate_cortex_BA24	$3.02x10^{-30}$	$1.83x10^{-35}$	$9.99x10^{-1}$	$4.82x10^{-6}$	$1.22x10^{-3}$
NRAD1, LACC1	3	Brain_Nucleus_accumbens_basal_ganglia	$1.03x10^{-34}$	$4.33x10^{-40}$	$9.99x10^{-1}$	$2.98x10^{-6}$	$1.22x10^{-3}$

NRAD1, LACC1	3	Brain_Hypothalamus	$2.15x10^{-32}$	$1.05x10^{-37}$	$9.99x10^{-1}$	$3.65x10^{-6}$	$1.22x10^{-3}$
NRAD1, LACC1	7	Brain_Caudate_basal_ganglia	$3.97x10^{-41}$	$4.61x10^{-46}$	$9.99x10^{-1}$	$1.04x10^{-5}$	$1.21x10^{-3}$
IGSF5	7	Brain_Amygdala	$7.83x10^{-1}$	$3.74x10^{-5}$	$2.15x10^{-1}$	$9.08x10^{-6}$	$1.21x10^{-3}$
C8Orf49	11	Heart_Atrial_Appendage	$3.28x10^{-25}$	$1.79x10^{-29}$	$9.99x10^{-1}$	$5.34x10^{-5}$	$1.21x10^{-3}$
NRAD1, LACC1	4	Whole_Blood	$5.57x10^{-87}$	$4.00x10^{-92}$	$9.99x10^{-1}$	$5.98x10^{-6}$	$1.21x10^{-3}$
C8Orf49	13	Minor_Salivary_Gland	$1.42x10^{-7}$	$3.41x10^{-12}$	$9.99x10^{-1}$	$2.27x10^{-5}$	$1.21x10^{-3}$
C8Orf49	10	Pancreas	$3.10x10^{-13}$	$3.98x10^{-18}$	$9.99x10^{-1}$	$1.16x10^{-5}$	$1.20x10^{-3}$
NRAD1, LACC1	6	Brain_Cerebellum	$1.91x10^{-49}$	$1.80x10^{-54}$	$9.99x10^{-1}$	$8.20x10^{-6}$	$1.20x10^{-3}$
NRAD1, LACC1	9	Pituitary	$1.88x10^{-51}$	$2.39x10^{-56}$	$9.99x10^{-1}$	$1.15x10^{-5}$	$1.20x10^{-3}$
NRAD1, LACC1	7	Skin_Not_Sun_Exposed_Suprapubic	$5.04x10^{-120}$	$2.05x10^{-124}$	$9.99x10^{-1}$	$3.95x10^{-5}$	$1.20x10^{-3}$
NRAD1, LACC1	4	Brain_Cortex	$5.14x10^{-35}$	$2.35x10^{-40}$	$9.99x10^{-1}$	$3.37x10^{-6}$	$1.20x10^{-3}$
TAS2R43, SMIM10L1, TAS2R14, TAS2R31	12	Brain_Amygdala	$1.20x10^{-3}$	$1.89x10^{-8}$	$9.98x10^{-1}$	$1.45x10^{-5}$	$1.20x10^{-3}$
CDH17	5	Skin_Sun_Exposed_Lower_leg	$1.81x10^{-1}$	$1.34x10^{-6}$	$8.18x10^{-1}$	$4.87x10^{-6}$	$1.19x10^{-3}$
NRAD1, LACC1	9	Adrenal_Gland	$1.52x10^{-35}$	$6.77x10^{-40}$	$9.99x10^{-1}$	$4.32x10^{-5}$	$1.19x10^{-3}$
NRAD1, LACC1	9	Liver	$5.86x10^{-39}$	$1.23x10^{-43}$	$9.99x10^{-1}$	$1.98x10^{-5}$	$1.19x10^{-3}$
NRAD1, LACC1	6	Thyroid	$1.61x10^{-88}$	$1.87x10^{-93}$	$9.99x10^{-1}$	$1.04x10^{-5}$	$1.19x10^{-3}$
NRAD1, LACC1	7	Colon_Sigmoid	$7.80x10^{-69}$	$1.46x10^{-73}$	$9.99x10^{-1}$	$1.75x10^{-5}$	$1.19x10^{-3}$
NRAD1, LACC1	6	Stomach	$4.57x10^{-57}$	$3.07x10^{-62}$	$9.99x10^{-1}$	$5.53x10^{-6}$	$1.19x10^{-3}$
NRAD1, LACC1	6	Brain_Cerebellar_Hemisphere	$6.11x10^{-38}$	$4.89x10^{-43}$	$9.99x10^{-1}$	$6.80x10^{-6}$	$1.19x10^{-3}$
NRAD1, LACC1	8	Artery_Tibial	$5.45x10^{-92}$	$2.28x10^{-96}$	$9.99x10^{-1}$	$4.06x10^{-5}$	$1.19x10^{-3}$
C8Orf49	10	Brain_Hippocampus	$2.37x10^{-8}$	$2.84x10^{-13}$	$9.99x10^{-1}$	$1.08x10^{-5}$	$1.19x10^{-3}$
NRAD1, LACC1	6	Brain_Hippocampus	$1.41x10^{-37}$	$9.95x10^{-43}$	$9.99x10^{-1}$	$5.86x10^{-6}$	$1.18x10^{-3}$
NRAD1, LACC1	5	Esophagus_Gastroesophageal_Junction	$1.19x10^{-51}$	$1.21x10^{-56}$	$9.99x10^{-1}$	$9.04x10^{-6}$	$1.18x10^{-3}$
NRAD1, LACC1	8	Adipose_Visceral_Omentum	$1.27x10^{-89}$	$1.58x10^{-94}$	$9.99x10^{-1}$	$1.12x10^{-5}$	$1.18x10^{-3}$
NRAD1, LACC1	3	Brain_Substantia_nigra	$2.26x10^{-14}$	$7.68x10^{-20}$	$9.99x10^{-1}$	$2.22x10^{-6}$	$1.18x10^{-3}$
NRAD1, LACC1	8	Cells_EBV-transformed_lymphocytes	$3.34x10^{-13}$	$5.59x10^{-18}$	$9.99x10^{-1}$	$1.55x10^{-5}$	$1.18x10^{-3}$
NRAD1, LACC1	9	Testis	$1.60x10^{-23}$	$2.13x10^{-28}$	$9.99x10^{-1}$	$1.21x10^{-5}$	$1.18x10^{-3}$
NRAD1, LACC1	7	Spleen	$5.56x10^{-51}$	$7.76x10^{-56}$	$9.99x10^{-1}$	$1.28x10^{-5}$	$1.17x10^{-3}$

NRAD1, LACC1	5	Kidney_Cortex	$1.48x10^{-6}$	$1.60x10^{-11}$	$9.99x10^{-1}$	$9.58x10^{-6}$	$1.17x10^{-3}$
NRAD1, LACC1	8	Small_Intestine_Terminal_Ileum	$1.58x10^{-22}$	$3.68x10^{-27}$	$9.99x10^{-1}$	$2.20x10^{-5}$	$1.17x10^{-3}$
NRAD1, LACC1	6	Minor_Salivary_Gland	$5.35x10^{-23}$	$4.97x10^{-28}$	$9.99x10^{-1}$	$8.16x10^{-6}$	$1.14x10^{-3}$
TAS2R43, SMIM10L1, TAS2R14, TAS2R31	30	Nerve_Tibial	$9.23x10^{-99}$	$3.58x10^{-103}$	$9.99x10^{-1}$	$3.76x10^{-5}$	$1.13x10^{-3}$
C8Orf49	11	Brain_Putamen_basal_ganglia	$1.25x10^{-6}$	$2.13x10^{-11}$	$9.99x10^{-1}$	$1.58x10^{-5}$	$1.12x10^{-3}$
C8Orf49	13	Adipose_Subcutaneous	$1.30x10^{-14}$	$2.43x10^{-19}$	$9.99x10^{-1}$	$1.75x10^{-5}$	$1.12x10^{-3}$
C8Orf49	7	Colon_Sigmoid	$5.91x10^{-7}$	$8.10x10^{-12}$	$9.99x10^{-1}$	$1.26x10^{-5}$	$1.11x10^{-3}$
IGSF5	9	Spleen	$4.92x10^{-18}$	$5.46x10^{-23}$	$9.99x10^{-1}$	$9.97x10^{-6}$	$1.11x10^{-3}$
TAS2R43, SMIM10L1, TAS2R14, TAS2R31	18	Whole_Blood	$3.92x10^{-142}$	$8.08x10^{-147}$	$9.99x10^{-1}$	$1.95x10^{-5}$	$1.11x10^{-3}$
C8Orf49	13	Small_Intestine_Terminal_Ileum	$5.86x10^{-7}$	$1.03x10^{-11}$	$9.99x10^{-1}$	$1.65x10^{-5}$	$1.10x10^{-3}$
IGSF5	8	Colon_Sigmoid	$6.06x10^{-41}$	$6.93x10^{-46}$	$9.99x10^{-1}$	$1.03x10^{-5}$	$1.10x10^{-3}$
IGSF5	9	Brain_Frontal_Cortex_BA9	$3.90x10^{-14}$	$5.79x10^{-19}$	$9.99x10^{-1}$	$1.37x10^{-5}$	$1.09x10^{-3}$
TAS2R43, SMIM10L1, TAS2R14, TAS2R31	23	Spleen	$5.29x10^{-43}$	$2.20x10^{-47}$	$9.99x10^{-1}$	$4.05x10^{-5}$	$1.09x10^{-3}$
C8Orf49	8	Artery_Tibial	$8.79x10^{-41}$	$9.41x10^{-46}$	$9.99x10^{-1}$	$9.61x10^{-6}$	$1.08x10^{-3}$
C8Orf49	12	Stomach	$7.29x10^{-9}$	$4.88x10^{-13}$	$9.99x10^{-1}$	$6.58x10^{-5}$	$1.07x10^{-3}$
C8Orf49	19	Thyroid	$4.88x10^{-28}$	$1.30x10^{-32}$	$9.99x10^{-1}$	$2.55x10^{-5}$	$1.05x10^{-3}$
C8Orf49	14	Prostate	$2.62x10^{-12}$	$5.39x10^{-17}$	$9.99x10^{-1}$	$1.95x10^{-5}$	$1.03x10^{-3}$
C8Orf49	14	Pituitary	$7.02x10^{-13}$	$1.70x10^{-17}$	$9.99x10^{-1}$	$2.32x10^{-5}$	$1.02x10^{-3}$
IGSF5	6	Stomach	$3.11x10^{-7}$	$2.87x10^{-12}$	$9.99x10^{-1}$	$8.18x10^{-6}$	$1.02x10^{-3}$
C8Orf49	8	Adipose_Visceral_Omentum	$3.45x10^{-10}$	$2.63x10^{-15}$	$9.99x10^{-1}$	$6.60x10^{-6}$	$1.01x10^{-3}$
C8Orf49	12	Nerve_Tibial	$1.33x10^{-47}$	$3.02x10^{-52}$	$9.99x10^{-1}$	$2.16x10^{-5}$	$1.01x10^{-3}$
C8Orf49	8	Ovary	$1.58x10^{-7}$	$1.68x10^{-12}$	$9.99x10^{-1}$	$9.65x10^{-6}$	$1.01x10^{-3}$
C8Orf49	11	Uterus	$2.29x10^{-3}$	$2.81x10^{-8}$	$9.97x10^{-1}$	$1.12x10^{-5}$	$9.85x10^{-4}$
TAS2R43, SMIM10L1, TAS2R14, TAS2R31	17	Cells_Cultured_fibroblasts	$3.70x10^{-55}$	$9.53x10^{-60}$	$9.99x10^{-1}$	$2.48x10^{-5}$	$9.85x10^{-4}$
IGSF5	6	Heart_Atrial_Appendage	$8.97x10^{-4}$	$1.36x10^{-8}$	$9.98x10^{-1}$	$1.42x10^{-5}$	$9.84x10^{-4}$
TAS2R43, SMIM10L1, TAS2R14, TAS2R31	8	Muscle_Skeletal	$1.18x10^{-62}$	$1.97x10^{-67}$	$9.99x10^{-1}$	$1.56x10^{-5}$	$9.78x10^{-4}$
TAS2R43, SMIM10L1, TAS2R14, TAS2R31	19	Artery_Coronary	$1.55x10^{-19}$	$1.80x10^{-23}$	$9.99x10^{-1}$	$1.15x10^{-4}$	$9.61x10^{-4}$
C8Orf49	15	Brain_Nucleus_accumbens_basal_ganglia	$2.12x10^{-12}$	$5.25x10^{-17}$	$9.99x10^{-1}$	$2.38x10^{-5}$	$9.49x10^{-4}$
IGSF5	7	Brain_Cortex	$3.15x10^{-4}$	$5.91x10^{-9}$	$9.99x10^{-1}$	$1.78x10^{-5}$	$9.43x10^{-4}$
C8Orf49	11	Artery_Aorta	$3.47x10^{-13}$	$5.27x10^{-18}$	$9.99x10^{-1}$	$1.42x10^{-5}$	$9.37x10^{-4}$

CDH17	5	Colon_Transverse	4.42×10^{-1}	2.57×10^{-6}	5.57×10^{-1}	2.31×10^{-6}	9.37×10^{-4}
C8Orf49	12	Brain_Hypothalamus	3.55×10^{-5}	5.78×10^{-10}	9.99×10^{-1}	1.53×10^{-5}	9.23×10^{-4}
CDH17	12	Testis	1.35×10^{-78}	2.94×10^{-83}	9.99×10^{-1}	2.09×10^{-5}	9.22×10^{-4}
TAS2R43, SMIM10L1, TAS2R14, TAS2R31	12	Brain_Nucleus_accumbens_basal_ganglia	2.95×10^{-8}	4.55×10^{-13}	9.99×10^{-1}	1.45×10^{-5}	9.03×10^{-4}
TAS2R43, SMIM10L1, TAS2R14, TAS2R31	14	Uterus	1.21×10^{-13}	1.70×10^{-18}	9.99×10^{-1}	1.32×10^{-5}	8.90×10^{-4}
C8Orf49	18	Brain_Caudate_basal_ganglia	8.95×10^{-8}	2.19×10^{-12}	9.99×10^{-1}	2.36×10^{-5}	8.88×10^{-4}
TAS2R43, SMIM10L1, TAS2R14, TAS2R31	15	Brain_Hypothalamus	9.31×10^{-20}	1.47×10^{-24}	9.99×10^{-1}	1.49×10^{-5}	8.85×10^{-4}
C8Orf49	14	Skin_Sun_Exposed_Lower_leg	1.87×10^{-73}	7.12×10^{-78}	9.99×10^{-1}	3.72×10^{-5}	8.83×10^{-4}
C8Orf49	10	Whole_Blood	1.72×10^{-18}	2.06×10^{-23}	9.99×10^{-1}	1.11×10^{-5}	8.71×10^{-4}
CDH17	4	Brain_Spinal_cord_cervical_c-1	9.45×10^{-1}	1.06×10^{-4}	5.42×10^{-2}	5.21×10^{-6}	8.70×10^{-4}
TAS2R43, SMIM10L1, TAS2R14, TAS2R31	13	Brain_Substantia_nigra	7.71×10^{-11}	1.72×10^{-15}	9.99×10^{-1}	2.14×10^{-5}	8.69×10^{-4}
C8Orf49	8	Brain_Spinal_cord_cervical_c-1	9.63×10^{-3}	8.26×10^{-8}	9.89×10^{-1}	7.62×10^{-6}	8.68×10^{-4}
C8Orf49	9	Kidney_Cortex	5.63×10^{-1}	1.16×10^{-5}	4.36×10^{-1}	8.13×10^{-6}	8.67×10^{-4}
TAS2R43, SMIM10L1, TAS2R14, TAS2R31	15	Brain_Caudate_basal_ganglia	4.91×10^{-23}	9.69×10^{-28}	9.99×10^{-1}	1.88×10^{-5}	8.60×10^{-4}
CDH17	6	Pancreas	5.37×10^{-1}	7.71×10^{-6}	4.62×10^{-1}	5.77×10^{-6}	8.58×10^{-4}
TAS2R43, SMIM10L1, TAS2R14, TAS2R31	20	Small_Intestine_Terminal_Ileum	5.34×10^{-30}	1.36×10^{-34}	9.99×10^{-1}	2.46×10^{-5}	8.52×10^{-4}
TAS2R43, SMIM10L1, TAS2R14, TAS2R31	18	Artery_Tibial	1.53×10^{-76}	3.36×10^{-81}	9.99×10^{-1}	2.11×10^{-5}	8.46×10^{-4}
TAS2R43, SMIM10L1, TAS2R14, TAS2R31	22	Brain_Cerebellum	7.08×10^{-41}	1.71×10^{-45}	9.99×10^{-1}	2.33×10^{-5}	8.44×10^{-4}
TAS2R43, SMIM10L1, TAS2R14, TAS2R31	20	Brain_Spinal_cord_cervical_c-1	2.11×10^{-12}	5.63×10^{-17}	9.99×10^{-1}	2.58×10^{-5}	8.43×10^{-4}
TAS2R43, SMIM10L1, TAS2R14, TAS2R31	15	Brain_Putamen_basal_ganglia	2.55×10^{-17}	7.79×10^{-22}	9.99×10^{-1}	2.97×10^{-5}	8.38×10^{-4}
TAS2R43, SMIM10L1,	18	Colon_Sigmoid	1.92×10^{-47}	3.77×10^{-52}	9.99×10^{-1}	1.88×10^{-5}	8.33×10^{-4}

TAS2R14, TAS2R31							
TAS2R43, SMIM10L1, TAS2R14, TAS2R31	16	Brain_Hippocampus	6.53×10^{-12}	1.20×10^{-16}	9.99×10^{-1}	1.75×10^{-5}	8.32×10^{-4}
C8Orf49	14	Esophagus_Mucosa	2.29×10^{-26}	8.00×10^{-31}	9.99×10^{-1}	3.41×10^{-5}	8.30×10^{-4}
TAS2R43, SMIM10L1, TAS2R14, TAS2R31	19	Minor_Salivary_Gland	1.47×10^{-14}	1.67×10^{-18}	9.99×10^{-1}	1.13×10^{-4}	8.28×10^{-4}
TAS2R43, SMIM10L1, TAS2R14, TAS2R31	12	Brain_Cortex	2.20×10^{-22}	2.71×10^{-27}	9.99×10^{-1}	1.15×10^{-5}	8.26×10^{-4}
TAS2R43, SMIM10L1, TAS2R14, TAS2R31	22	Esophagus_Muscularis	4.93×10^{-73}	1.15×10^{-77}	9.99×10^{-1}	2.25×10^{-5}	8.24×10^{-4}
TAS2R43, SMIM10L1, TAS2R14, TAS2R31	21	Brain_Cerebellar_Hemisphere	2.72×10^{-35}	6.72×10^{-40}	9.99×10^{-1}	2.38×10^{-5}	8.24×10^{-4}
TAS2R43, SMIM10L1, TAS2R14, TAS2R31	21	Artery_Aorta	2.03×10^{-42}	5.41×10^{-47}	9.99×10^{-1}	2.57×10^{-5}	8.19×10^{-4}
TAS2R43, SMIM10L1, TAS2R14, TAS2R31	25	Adipose_Visceral_Omentum	4.34×10^{-74}	1.33×10^{-78}	9.99×10^{-1}	2.98×10^{-5}	8.18×10^{-4}
TAS2R43, SMIM10L1, TAS2R14, TAS2R31	24	Ovary	1.18×10^{-24}	3.03×10^{-29}	9.99×10^{-1}	2.49×10^{-5}	8.16×10^{-4}
IGSF5	8	Muscle_Skeletal	1.03×10^{-41}	4.24×10^{-46}	9.99×10^{-1}	4.04×10^{-5}	8.12×10^{-4}
C8Orf49	19	Skin_Not_Sun_Exposed_Suprapubic	1.59×10^{-36}	8.65×10^{-41}	9.99×10^{-1}	5.34×10^{-5}	8.11×10^{-4}
TAS2R43, SMIM10L1, TAS2R14, TAS2R31	14	Heart_Atrial_Appendage	3.24×10^{-52}	5.43×10^{-57}	9.99×10^{-1}	1.59×10^{-5}	8.05×10^{-4}
TAS2R43, SMIM10L1, TAS2R14, TAS2R31	19	Esophagus_Mucosa	1.05×10^{-60}	1.94×10^{-65}	9.99×10^{-1}	1.76×10^{-5}	8.05×10^{-4}
IGSF5	9	Esophagus_Mucosa	1.20×10^{-62}	3.71×10^{-67}	9.99×10^{-1}	3.00×10^{-5}	8.04×10^{-4}
IGSF5	8	Skin_Not_Sun_Exposed_Suprapubic	1.51×10^{-80}	4.43×10^{-85}	9.99×10^{-1}	2.85×10^{-5}	8.03×10^{-4}
TAS2R43, SMIM10L1, TAS2R14, TAS2R31	16	Heart_Left_Ventricle	9.76×10^{-55}	2.09×10^{-59}	9.99×10^{-1}	2.05×10^{-5}	8.02×10^{-4}

TAS2R43, SMIM10L1, TAS2R14, TAS2R31	19	Esophagus_Gastroesophageal_Junction	$4.05x10^{-44}$	$1.20x10^{-48}$	$9.99x10^{-1}$	$2.88x10^{-5}$	$8.01x10^{-4}$
TAS2R43, SMIM10L1, TAS2R14, TAS2R31	18	Vagina	$1.30x10^{-17}$	$3.69x10^{-22}$	$9.99x10^{-1}$	$2.75x10^{-5}$	$7.99x10^{-4}$
C8Orf49	13	Cells_Cultured_fibroblasts	$1.65x10^{-34}$	$2.42x10^{-39}$	$9.99x10^{-1}$	$1.38x10^{-5}$	$7.99x10^{-4}$
IGSF5	8	Skin_Sun_Exposed_Lower_leg	$2.17x10^{-87}$	$5.10x10^{-92}$	$9.99x10^{-1}$	$2.27x10^{-5}$	$7.99x10^{-4}$
TAS2R43, SMIM10L1, TAS2R14, TAS2R31	17	Colon_Transverse	$2.77x10^{-43}$	$5.87x10^{-48}$	$9.99x10^{-1}$	$2.04x10^{-5}$	$7.98x10^{-4}$
TAS2R43, SMIM10L1, TAS2R14, TAS2R31	24	Testis	$1.06x10^{-26}$	$3.05x10^{-31}$	$9.99x10^{-1}$	$2.81x10^{-5}$	$7.98x10^{-4}$
TAS2R43, SMIM10L1, TAS2R14, TAS2R31	12	Pancreas	$5.50x10^{-40}$	$5.93x10^{-45}$	$9.99x10^{-1}$	$9.97x10^{-6}$	$7.97x10^{-4}$
TAS2R43, SMIM10L1, TAS2R14, TAS2R31	21	Breast_Mammary_Tissue	$8.29x10^{-52}$	$2.18x10^{-56}$	$9.99x10^{-1}$	$2.54x10^{-5}$	$7.96x10^{-4}$
TAS2R43, SMIM10L1, TAS2R14, TAS2R31	21	Lung	$5.17x10^{-81}$	$1.26x10^{-85}$	$9.99x10^{-1}$	$2.36x10^{-5}$	$7.96x10^{-4}$
TAS2R43, SMIM10L1, TAS2R14, TAS2R31	22	Adipose_Subcutaneous	$1.08x10^{-88}$	$2.71x10^{-93}$	$9.99x10^{-1}$	$2.44x10^{-5}$	$7.95x10^{-4}$
TAS2R43, SMIM10L1, TAS2R14, TAS2R31	21	Skin_Not_Sun_Exposed_Suprapubic	$6.24x10^{-72}$	$1.39x10^{-76}$	$9.99x10^{-1}$	$2.15x10^{-5}$	$7.94x10^{-4}$
TAS2R43, SMIM10L1, TAS2R14, TAS2R31	19	Prostate	$3.63x10^{-32}$	$8.05x10^{-37}$	$9.99x10^{-1}$	$2.14x10^{-5}$	$7.94x10^{-4}$
IGSF5	6	Brain_Nucleus_accumbens_basal_ganglia	$4.08x10^{-1}$	$4.30x10^{-6}$	$5.92x10^{-1}$	$5.45x10^{-6}$	$7.94x10^{-4}$
TAS2R43, SMIM10L1, TAS2R14, TAS2R31	17	Liver	$1.66x10^{-29}$	$6.62x10^{-34}$	$9.99x10^{-1}$	$3.90x10^{-5}$	$7.94x10^{-4}$
TAS2R43, SMIM10L1, TAS2R14, TAS2R31	25	Thyroid	$7.81x10^{-101}$	$2.09x10^{-105}$	$9.99x10^{-1}$	$2.60x10^{-5}$	$7.93x10^{-4}$

TAS2R43, SMIM10L1, TAS2R14, TAS2R31	13	Stomach	$5.38x10^{-38}$	$7.15x10^{-43}$	$9.99x10^{-1}$	$1.25x10^{-5}$	$7.92x10^{-4}$
TAS2R43, SMIM10L1, TAS2R14, TAS2R31	16	Cells_EBV-transformed_lymphocytes	$3.85x10^{-20}$	$6.45x10^{-25}$	$9.99x10^{-1}$	$1.60x10^{-5}$	$7.92x10^{-4}$
TAS2R43, SMIM10L1, TAS2R14, TAS2R31	17	Adrenal_Gland	$1.95x10^{-27}$	$5.02x10^{-32}$	$9.99x10^{-1}$	$2.50x10^{-5}$	$7.91x10^{-4}$
C8Orf49	5	Vagina	$9.77x10^{-2}$	$5.75x10^{-7}$	$9.02x10^{-1}$	$4.52x10^{-6}$	$7.89x10^{-4}$
IGSF5	5	Colon_Transverse	$2.73x10^{-31}$	$2.08x10^{-36}$	$9.99x10^{-1}$	$6.82x10^{-6}$	$7.88x10^{-4}$
C8Orf49	10	Brain_Substantia_nigra	$1.21x10^{-2}$	$1.95x10^{-7}$	$9.87x10^{-1}$	$1.51x10^{-5}$	$7.86x10^{-4}$
IGSF5	9	Liver	$3.86x10^{-5}$	$7.99x10^{-10}$	$9.99x10^{-1}$	$1.99x10^{-5}$	$7.85x10^{-4}$
IGSF5	6	Small_Intestine_Terminal_Ileum	$1.30x10^{-12}$	$9.70x10^{-18}$	$9.99x10^{-1}$	$6.70x10^{-6}$	$7.84x10^{-4}$
CDH17	9	Heart_Atrial_Appendage	$5.83x10^{-3}$	$1.00x10^{-7}$	$9.93x10^{-1}$	$1.63x10^{-5}$	$7.83x10^{-4}$
NRAD1, LACC1	7	Prostate	$2.79x10^{-21}$	$2.22x10^{-26}$	$9.99x10^{-1}$	$7.17x10^{-6}$	$7.76x10^{-4}$
IGSF5	7	Testis	$6.67x10^{-26}$	$7.31x10^{-31}$	$9.99x10^{-1}$	$1.02x10^{-5}$	$7.67x10^{-4}$
CDH17	6	Brain_Cortex	$6.44x10^{-1}$	$5.16x10^{-6}$	$3.55x10^{-1}$	$2.20x10^{-6}$	$6.47x10^{-4}$
IGSF5	5	Uterus	$8.08x10^{-1}$	$8.92x10^{-6}$	$1.92x10^{-1}$	$1.58x10^{-6}$	$5.33x10^{-4}$
CDH17	3	Cells_EBV-transformed_lymphocytes	$6.31x10^{-1}$	$3.12x10^{-6}$	$3.68x10^{-1}$	$1.29x10^{-6}$	$5.29x10^{-4}$
CDH17	4	Vagina	$9.78x10^{-1}$	$4.31x10^{-5}$	$2.16x10^{-2}$	$4.38x10^{-7}$	$5.15x10^{-4}$
CDH17	6	Cells_Cultured_fibroblasts	$8.03x10^{-1}$	$8.02x10^{-6}$	$1.97x10^{-1}$	$1.67x10^{-6}$	$2.92x10^{-4}$
CDH17	5	Artery_Tibial	$9.05x10^{-1}$	$6.81x10^{-6}$	$9.51x10^{-2}$	$5.06x10^{-7}$	$2.10x10^{-4}$
CDH17	5	Prostate	$8.27x10^{-1}$	$1.94x10^{-5}$	$1.73x10^{-1}$	$3.86x10^{-6}$	$2.05x10^{-4}$
CDH17	2	Brain_Anterior_cingulate_cortex_BA24	$8.72x10^{-1}$	$2.63x10^{-6}$	$1.28x10^{-1}$	$1.81x10^{-7}$	$2.04x10^{-4}$
CDH17	3	Colon_Sigmoid	$8.19x10^{-1}$	$3.38x10^{-6}$	$1.80x10^{-1}$	$5.71x10^{-7}$	$1.74x10^{-4}$
IGSF5	5	Brain_Anterior_cingulate_cortex_BA24	$8.69x10^{-1}$	$7.18x10^{-6}$	$1.31x10^{-1}$	$9.23x10^{-7}$	$1.55x10^{-4}$
CDH17	6	Brain_Hypothalamus	$8.85x10^{-1}$	$8.03x10^{-6}$	$1.15x10^{-1}$	$9.03x10^{-7}$	$1.41x10^{-4}$
CDH17	4	Adrenal_Gland	$9.46x10^{-1}$	$9.58x10^{-6}$	$5.39x10^{-2}$	$4.08x10^{-7}$	$1.38x10^{-4}$
CDH17	3	Skin_Not_Sun_Exposed_Suprapubic	$8.80x10^{-1}$	$4.93x10^{-6}$	$1.20x10^{-1}$	$5.47x10^{-7}$	$1.26x10^{-4}$
CDH17	6	Brain_Hippocampus	$9.11x10^{-1}$	$6.99x10^{-6}$	$8.88x10^{-2}$	$5.61x10^{-7}$	$1.20x10^{-4}$
CDH17	3	Brain_Putamen_basal_ganglia	$9.44x10^{-1}$	$9.97x10^{-6}$	$5.54x10^{-2}$	$4.69x10^{-7}$	$1.16x10^{-4}$
CDH17	5	Brain_Cerebellum	$9.53x10^{-1}$	$1.14x10^{-5}$	$4.66x10^{-2}$	$4.51x10^{-7}$	$1.07x10^{-4}$
CDH17	2	Brain_Cerebellar_Hemisphere	$9.21x10^{-1}$	$2.34x10^{-6}$	$7.90x10^{-2}$	$9.73x10^{-8}$	$1.03x10^{-4}$
CDH17	4	Breast_Mammary_Tissue	$9.08x10^{-1}$	$4.02x10^{-6}$	$9.17x10^{-2}$	$3.13x10^{-7}$	$9.28x10^{-5}$
IGSF5	4	Kidney_Cortex	$9.66x10^{-1}$	$7.01x10^{-6}$	$3.36x10^{-2}$	$1.60x10^{-7}$	$8.34x10^{-5}$
IGSF5	4	Brain_Substantia_nigra	$9.47x10^{-1}$	$6.33x10^{-6}$	$5.29x10^{-2}$	$2.78x10^{-7}$	$7.46x10^{-5}$
CDH17	2	/Lung	$9.56x10^{-1}$	$2.93x10^{-6}$	$4.38x10^{-2}$	$6.99x10^{-8}$	$6.44x10^{-5}$
CDH17	2	Brain_Nucleus_accumbens_basal_ganglia	$9.89x10^{-1}$	$9.22x10^{-6}$	$1.12x10^{-2}$	$5.69x10^{-8}$	$4.71x10^{-5}$
CDH17	4	Esophagus_Gastroesophageal_Junction	$9.46x10^{-1}$	$3.38x10^{-6}$	$5.36x10^{-2}$	$1.45x10^{-7}$	$4.69x10^{-5}$
IGSF5	1	Cells_EBV-transformed_lymphocytes	$9.86x10^{-1}$	$3.01x10^{-6}$	$1.37x10^{-2}$	0	$4.18x10^{-5}$
CDH17	4	Small_Intestine_Terminal_Ileum	$9.66x10^{-1}$	$4.35x10^{-6}$	$3.37x10^{-2}$	$1.13x10^{-7}$	$3.89x10^{-5}$
CDH17	3	Brain_Caudate_basal_ganglia	$9.77x10^{-1}$	$7.00x10^{-6}$	$2.27x10^{-2}$	$1.29x10^{-7}$	$3.39x10^{-5}$
CDH17	8	Kidney_Cortex	$9.74x10^{-1}$	$1.13x10^{-5}$	$2.62x10^{-2}$	$2.75x10^{-7}$	$2.98x10^{-5}$
CDH17	4	Pituitary	$9.74x10^{-1}$	$4.34x10^{-6}$	$2.60x10^{-2}$	$8.62x10^{-8}$	$2.97x10^{-5}$
CDH17	4	Uterus	$9.80x10^{-1}$	$5.53x10^{-6}$	$2.01x10^{-2}$	$8.46x10^{-8}$	$2.86x10^{-5}$
CDH17	4	Artery_Aorta	$9.69x10^{-1}$	$3.92x10^{-6}$	$3.12x10^{-2}$	$9.82x10^{-8}$	$2.80x10^{-5}$

CDH17	4	Liver	$9.92x10^{-1}$	$1.18x10^{-5}$	$8.01x10^{-3}$	$6.75x10^{-8}$	$2.76x10^{-5}$
CDH17	2	Esophagus_Muscularis	$9.89x10^{-1}$	$4.09x10^{-6}$	$1.06x10^{-2}$	$2.07x10^{-8}$	$2.30x10^{-5}$
CDH17	1	Minor_Salivary_Gland	$9.90x10^{-1}$	$2.19x10^{-6}$	$9.54x10^{-3}$	0	$2.11x10^{-5}$
CDH17	3	Brain_Substantia_nigra	$9.87x10^{-1}$	$4.67x10^{-6}$	$1.35x10^{-2}$	$4.30x10^{-8}$	$2.07x10^{-5}$
CDH17	3	Muscle_Skeletal	$9.78x10^{-1}$	$3.56x10^{-6}$	$2.18x10^{-2}$	$5.87x10^{-8}$	$2.06x10^{-5}$
CDH17	3	Whole_Blood	$9.93x10^{-1}$	$9.30x10^{-6}$	$6.77x10^{-3}$	$4.53x10^{-8}$	$1.81x10^{-5}$
CDH17	2	Brain_Amygdala	$9.90x10^{-1}$	$2.33x10^{-6}$	$9.81x10^{-3}$	$1.14x10^{-8}$	$1.17x10^{-5}$
CDH17	2	Ovary	$9.95x10^{-1}$	$4.01x10^{-6}$	$4.50x10^{-3}$	$1.05x10^{-8}$	$7.63x10^{-6}$
CDH17	2	Adipose_Visceral_Omentum	$9.92x10^{-1}$	$2.29x10^{-6}$	$8.45x10^{-3}$	$1.21x10^{-8}$	$7.46x10^{-6}$
CDH17	1	Brain_Frontal_Cortex_BA9	$9.94x10^{-1}$	$8.92x10^{-7}$	$5.54x10^{-3}$	0	$4.97x10^{-6}$

Figures

A.1

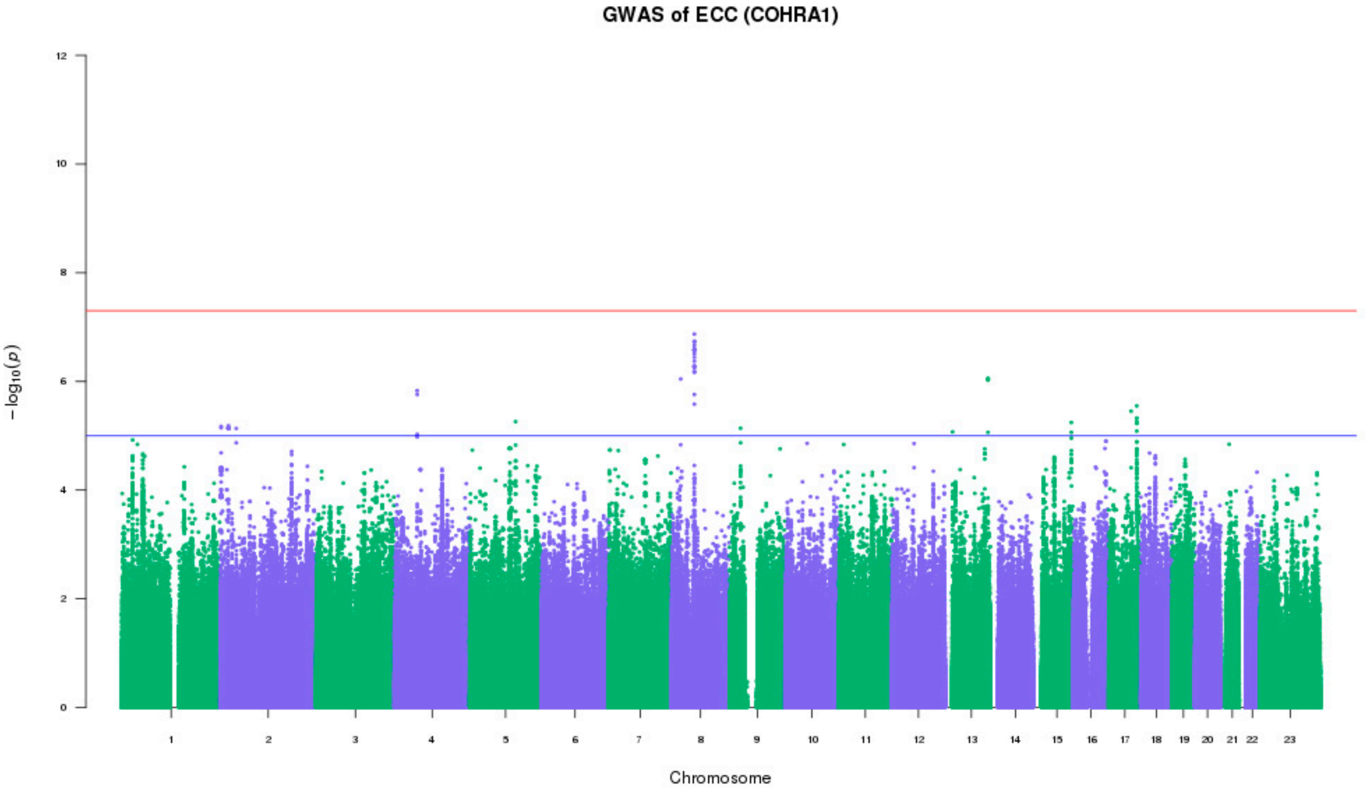

A.2

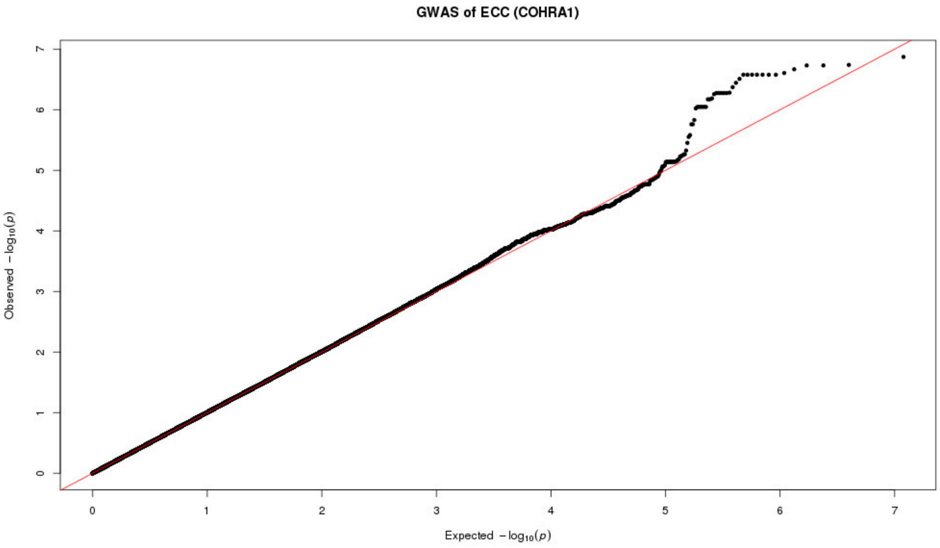
GWAS of ECC (COHRA1)
Observed −log10(p)
Expected −log10(p)

B.1

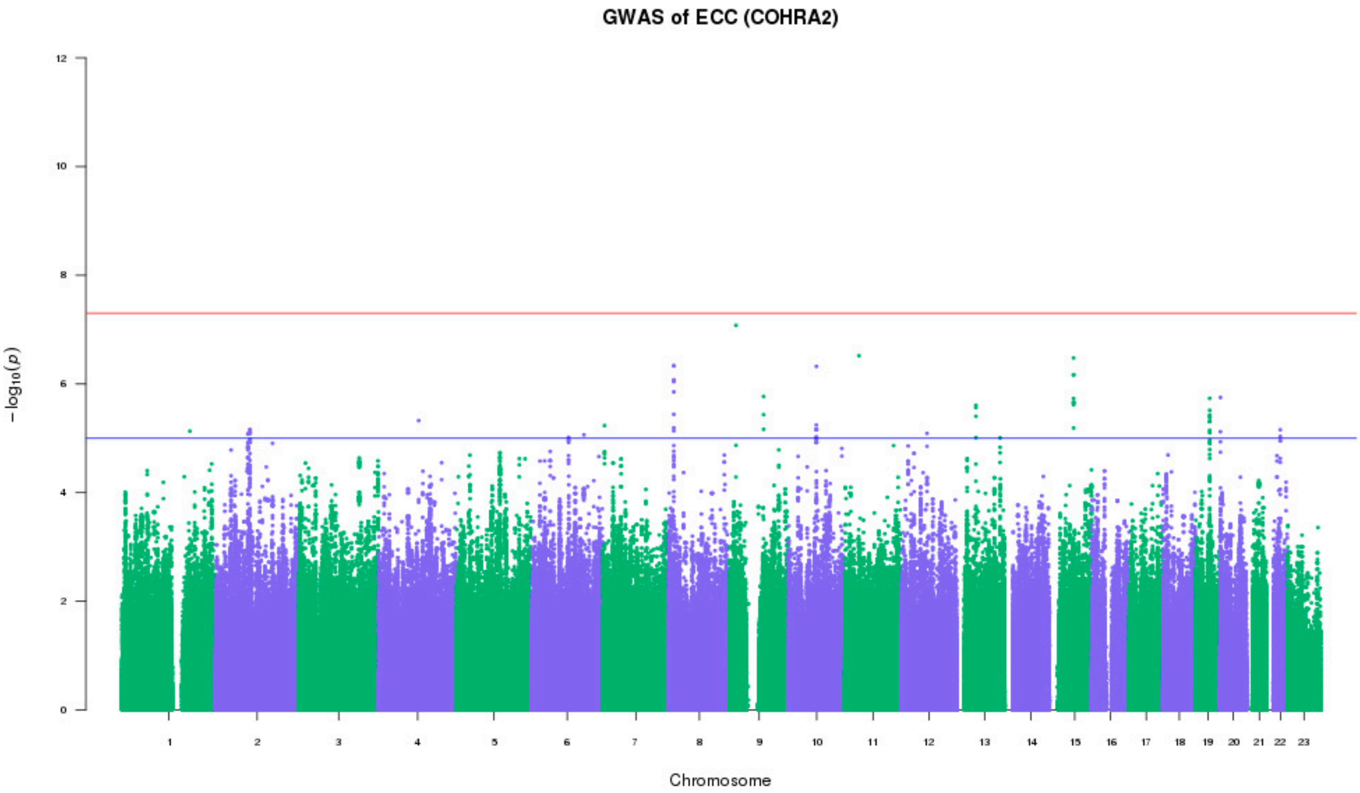
GWAS of ECC (COHRA2)
−log10(p)
Chromosome

B.2

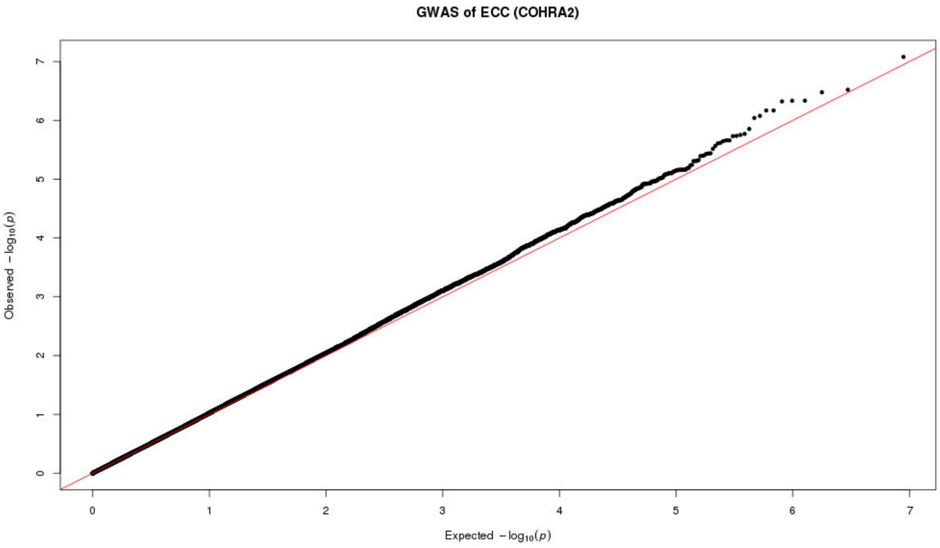
GWAS of ECC (COHRA2)
Observed $-\log_{10}(p)$
Expected $-\log_{10}(p)$
0
1
2
3
4
5
6
7

C.1

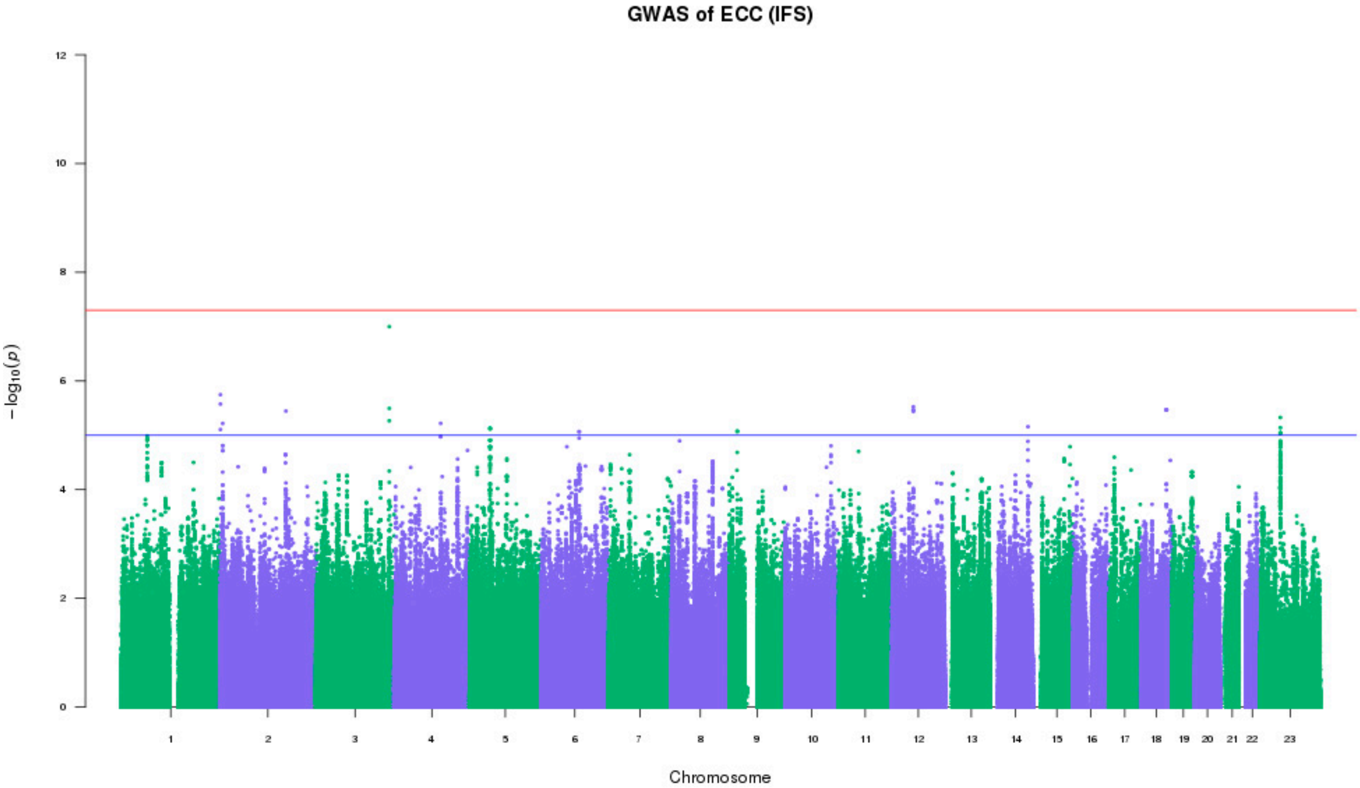
GWAS of ECC (IFS)
$-\log_{10}(p)$
Chromosome
0
2
4
6
8
10
12
1
2
3
4
5
6
7
8
9
10
11
12
13
14
15
16
17
18
19
20
21
22
23

C.2

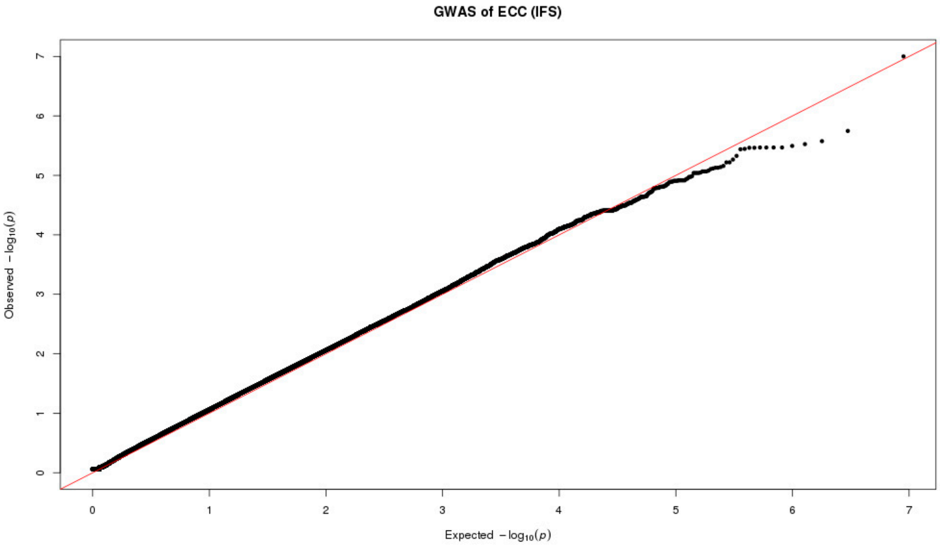
GWAS of ECC (IFS)
Observed $-\log_{10}(p)$
Expected $-\log_{10}(p)$
0
1
2
3
4
5
6
7

D.1

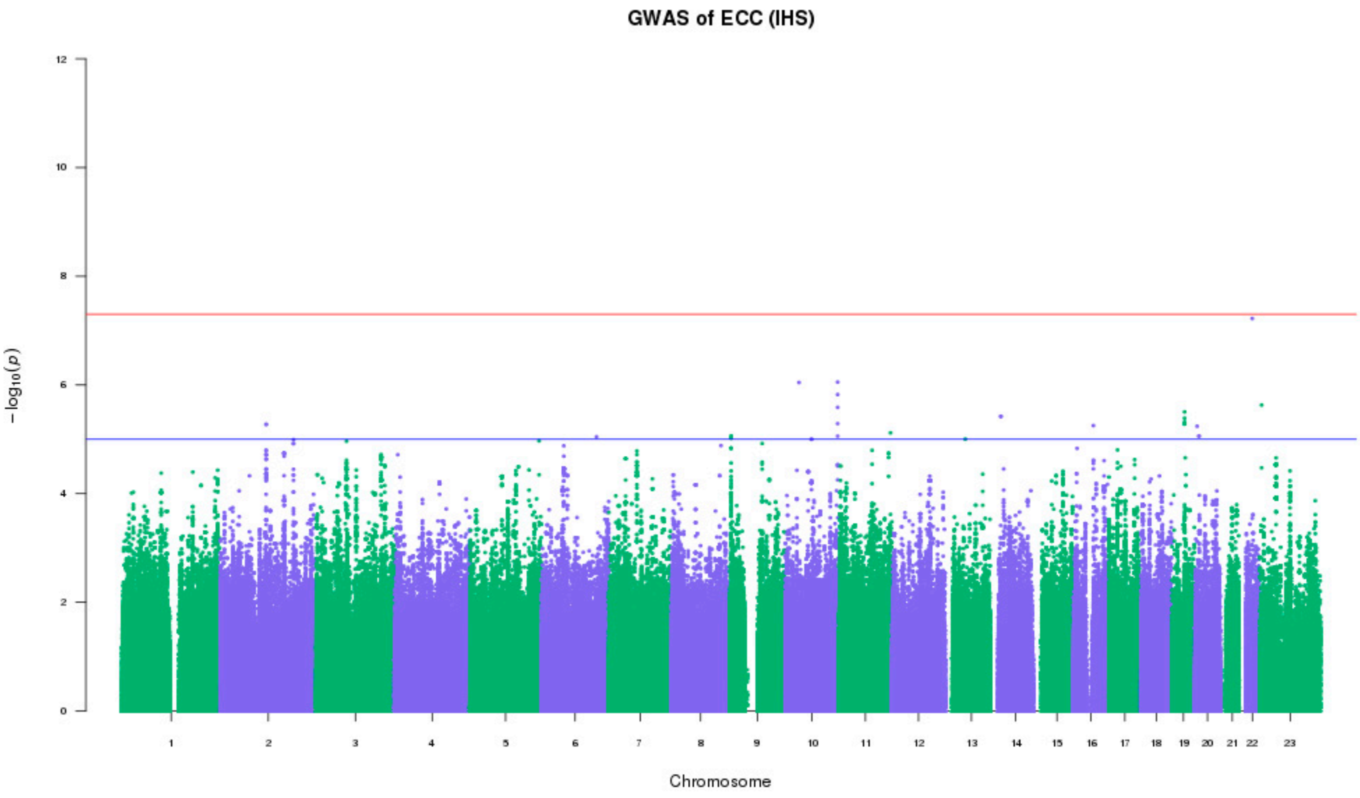
GWAS of ECC (IHS)
$-\log_{10}(p)$
Chromosome
0
2
4
6
8
10
12
1 2 3 4 5 6 7 8 9 10 11 12 13 14 15 16 17 18 19 20 21 22 23

D.2

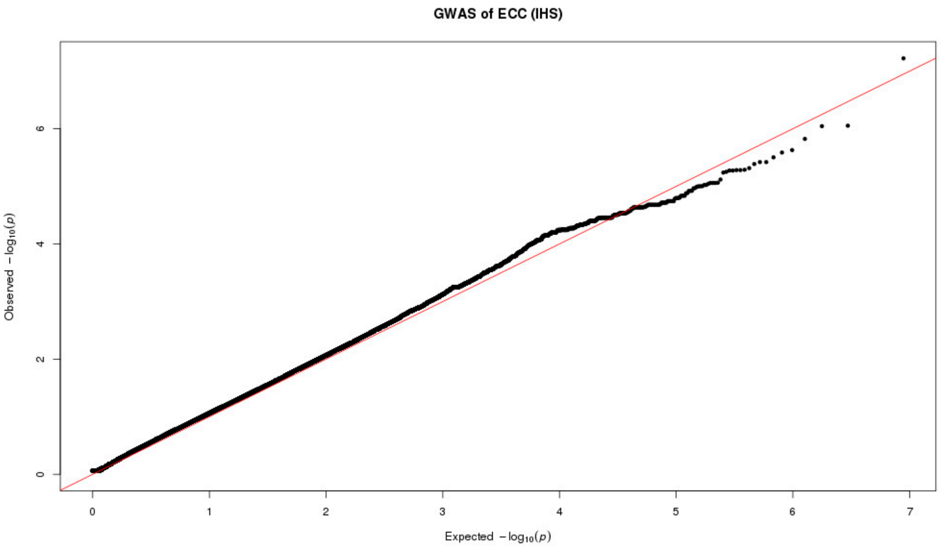
GWAS of ECC (IHS)
Observed −log10(p)
Expected −log10(p)
0
2
4
6
0
1
2
3
4
5
6
7

E.1

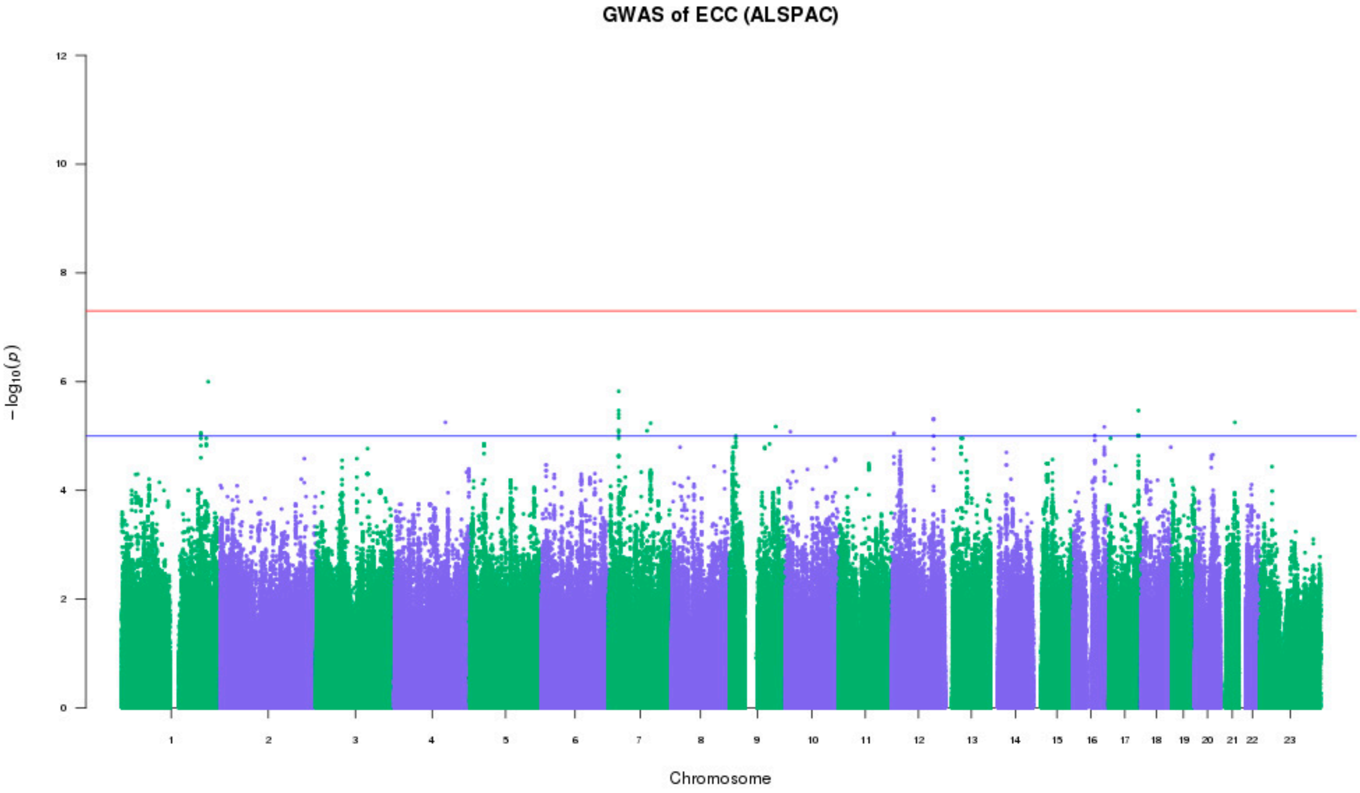
GWAS of ECC (ALSPAC)
−log10(p)
Chromosome
0
2
4
6
8
10
12
1
2
3
4
5
6
7
8
9
10
11
12
13
14
15
16
17
18
19
20
21
22
23

E.2

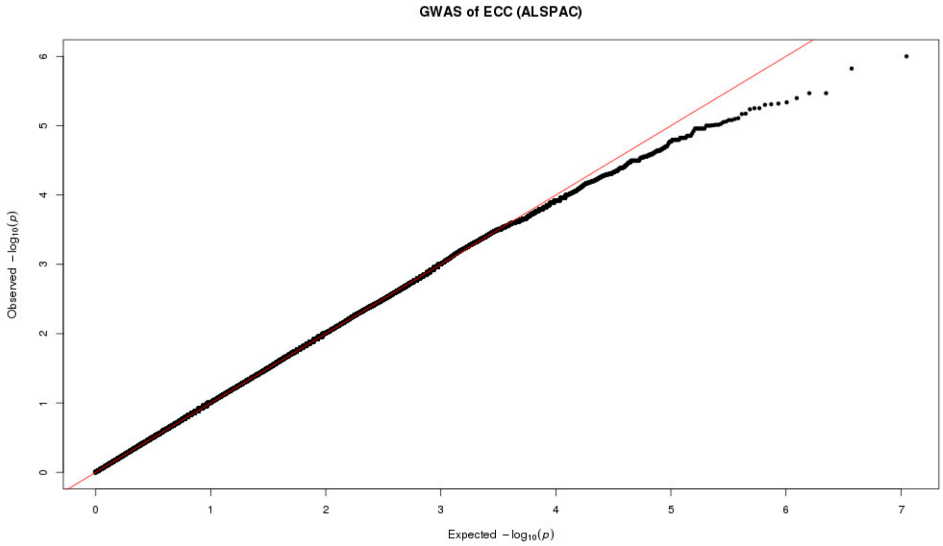

Figure S1. Manhattan and quantile-quantile (qq) plots of genome-wide association studies (GWASs) of early childhood caries (ECC) in COHRA1, COHRA2, IFS, IHS, and ALSPAC cohorts. In the Manhattan plots, the horizontal red line represents the genome-wide significance threshold (p=5×10^{-8}), and the horizontal blue line is the suggestive significance threshold (p=1×10^{-5}). Each point represents a variant tested for association. The x-axis is the genomic position of the corresponding variant, and the y-axis is the negative logarithm of the association p-value. In the qq plots, the negative logarithms of observed p-values of the GWAS (y-axis) are plotted against the expected p-values from the null hypothesis (x-axis). A.1, Manhattan plot of GWAS of ECC in COHRA1. No loci were associated at genome-wide significance. A.2, qq plot of GWAS of ECC in COHRA1. The inflation factor Λ is 1.03. B.1, Manhattan plot of GWAS of ECC in COHRA2. No loci were associated at genome-wide significance. B.2, qq plot of GWAS of ECC in COHRA2. Λ = 1.09. C.1, Manhattan plot of GWAS of ECC in Iowa Fluoride Study. No loci were associated at genome-wide significance. C.2, qq plot of GWAS of ECC in Iowa Fluoride Study. Λ = 0.88. D.1, Manhattan plot of GWAS of ECC in Iowa Head Start. No loci were associated at genome-wide significance. D.2, qq plot of GWAS of ECC in Iowa Head Start. Λ = 0.88. E.1, Manhattan plot of GWAS of ECC in ALSPAC. No loci were associated at genome-wide significance. E.2, qq plot of GWAS of ECC in ALSPAC. Λ = 1.0.

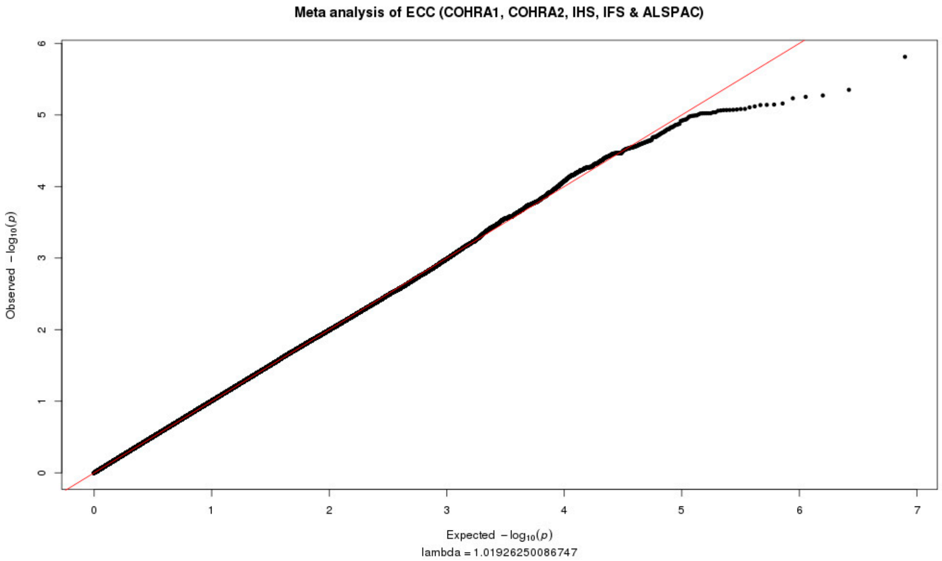

Figure S2. Quantile-quantile (qq) plot of the meta-analysis of early childhood caries (ECC) genome-wide association studies (GWASs.) The negative logarithms of observed p-values of the GWAS (y-axis) are plotted against the expected p-values from the null hypothesis (x-axis). The inflation factor λ is 1.01.

A

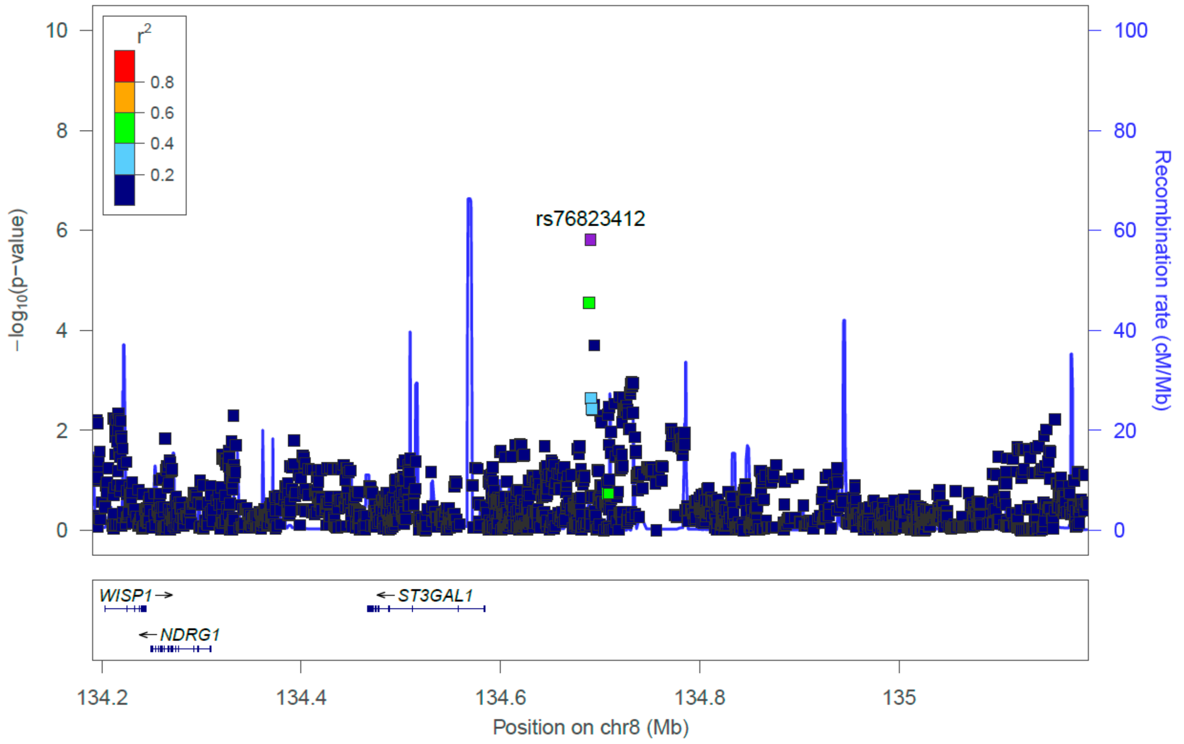
rs76823412
r2
0.8
0.6
0.4
0.2
−log10(p−value)
Recombination rate (cM/Mb)
WISP1
NDRG1
ST3GAL1
134.2
134.4
134.6
134.8
135
Position on chr8 (Mb)

B

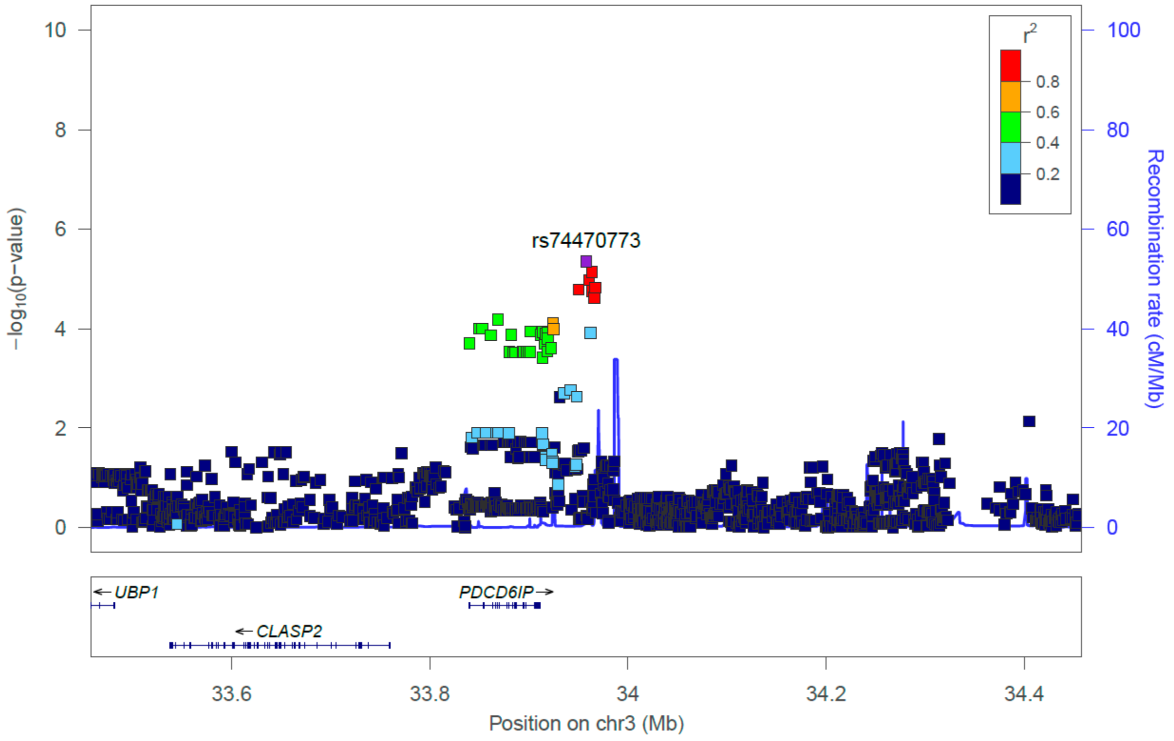
rs74470773
r2
0.8
0.6
0.4
0.2
−log10(p−value)
Recombination rate (cM/Mb)
UBP1
CLASP2
PDCD6IP
33.6
33.8
34
34.2
34.4
Position on chr3 (Mb)

C

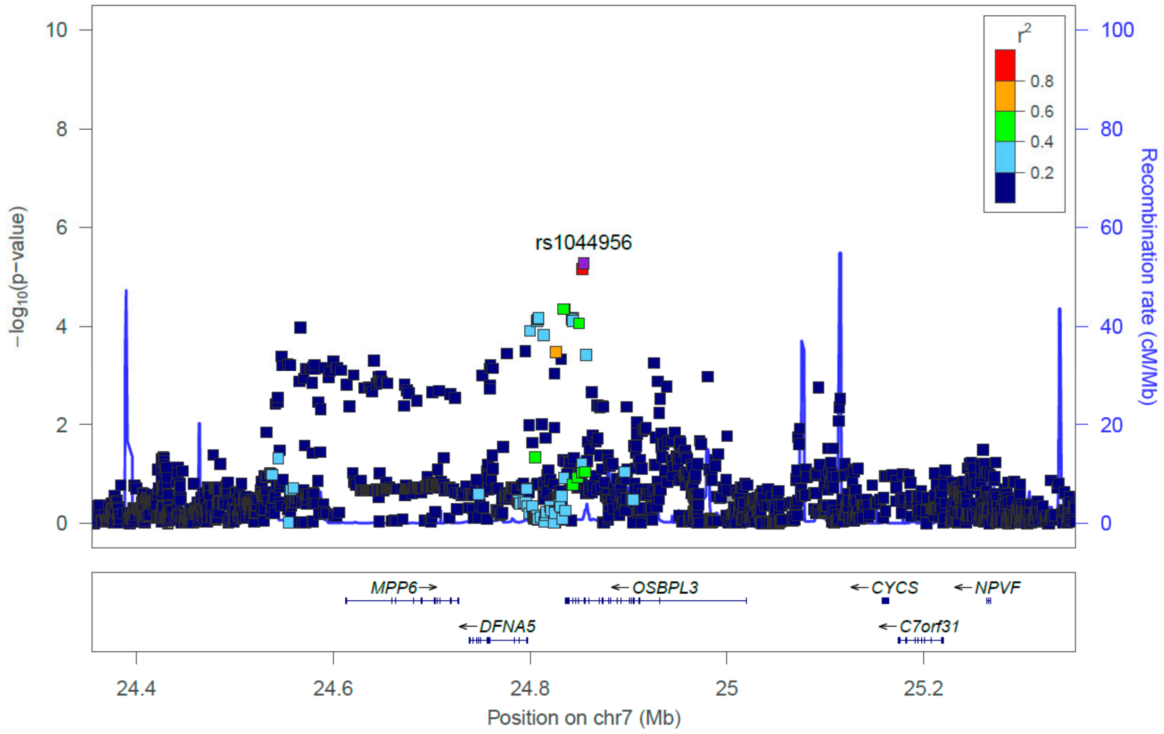
rs1044956
r2
0.8
0.6
0.4
0.2
−log10(p−value)
Recombination rate (cM/Mb)
MPP6
OSBPL3
CYCS
NPVF
DFNA5
C7orf31
Position on chr7 (Mb)
24.4
24.6
24.8
25
25.2

D

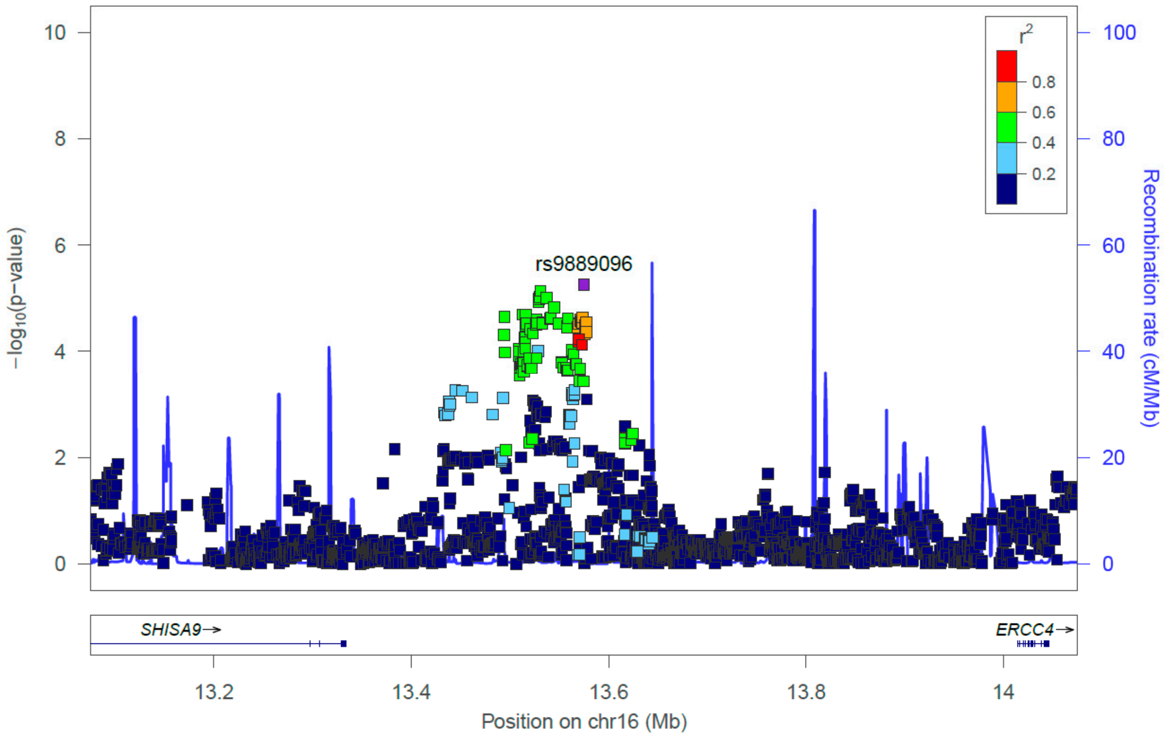
rs9889096
r2
0.8
0.6
0.4
0.2
−log10(p−value)
Recombination rate (cM/Mb)
SHISA9
ERCC4
Position on chr16 (Mb)
13.2
13.4
13.6
13.8
14

E

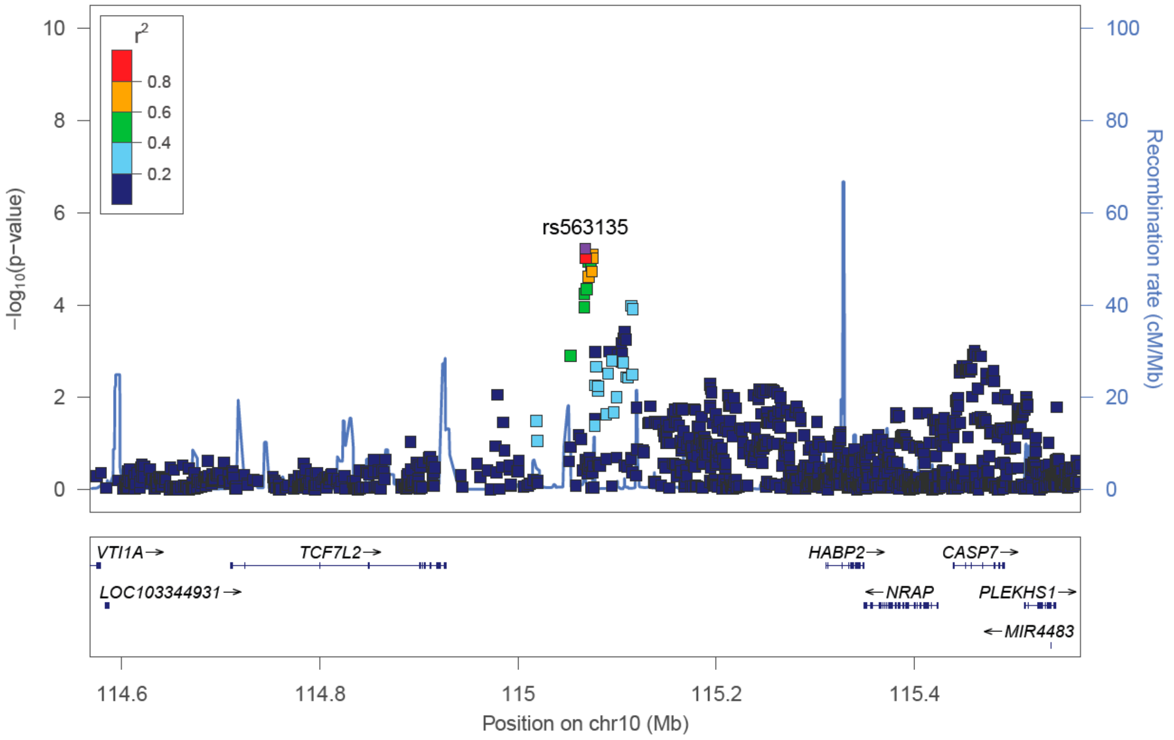
r2
0.8
0.6
0.4
0.2
rs563135
−log10(p−value)
Recombination rate (cM/Mb)
VTI1A→
TCF7L2→
HABP2→
CASP7→
LOC103344931→
←NRAP
PLEKHS1→
←MIR4483
114.6
114.8
115
115.2
115.4
Position on chr10 (Mb)

F

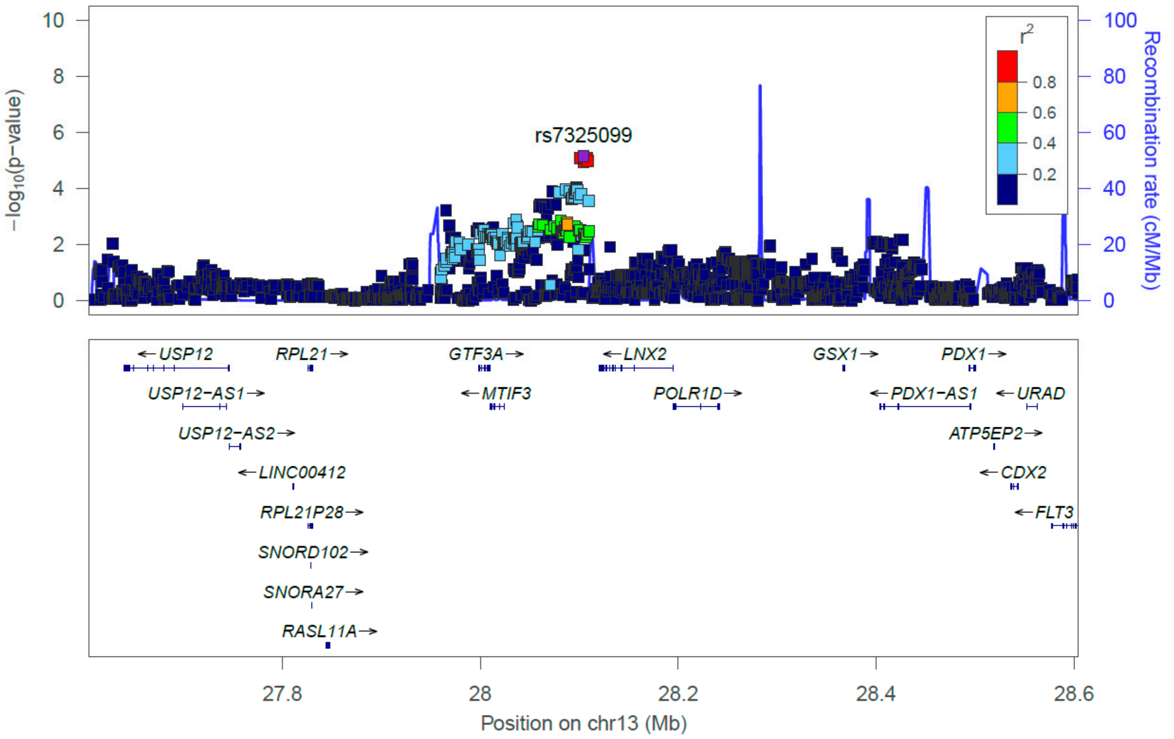
r2
0.8
0.6
0.4
0.2
rs7325099
−log10(p−value)
Recombination rate (cM/Mb)
←USP12
RPL21→
GTF3A→
←LNX2
GSX1→
PDX1→
USP12−AS1→
←MTIF3
POLR1D→
←PDX1−AS1
←URAD
USP12−AS2→
ATP5EP2→
←LINC00412
←CDX2
RPL21P28→
←FLT3
SNORD102→
SNORA27→
RASL11A→
27.8
28
28.2
28.4
28.6
Position on chr13 (Mb)

G

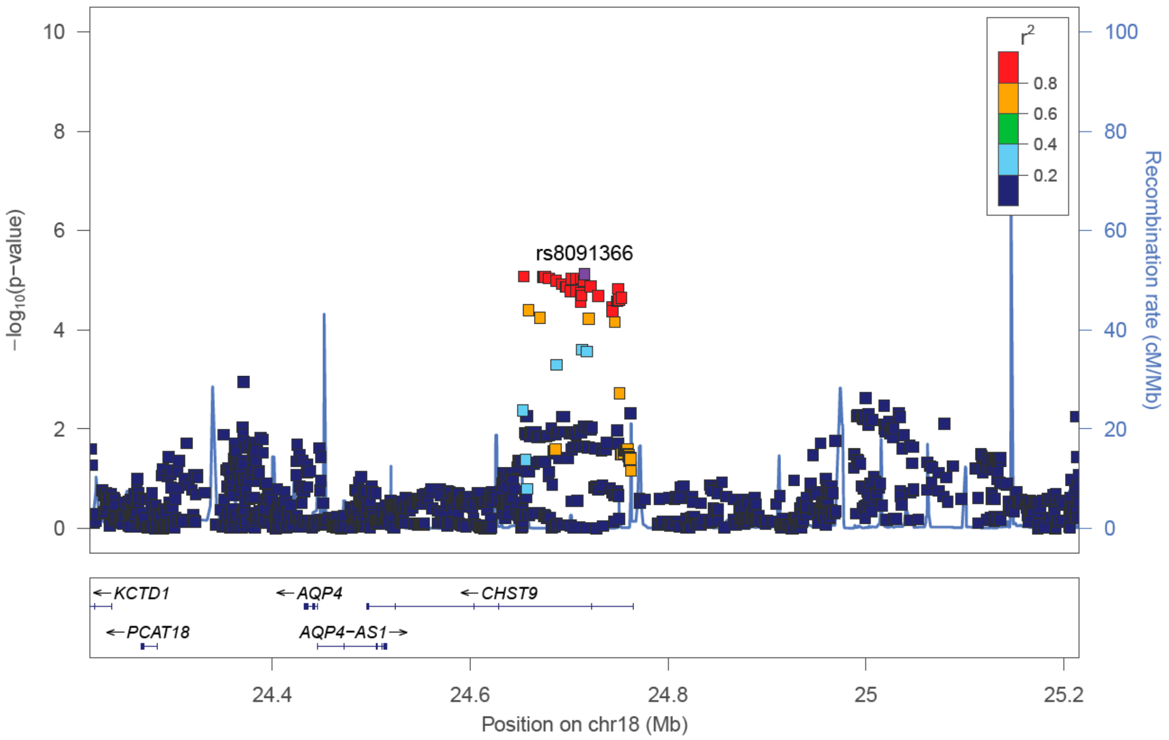

Figure S3. LocusZoom plots of suggestively significantly associated (p-value < 1x10^{-5}) loci from the meta-analysis of early childhood caries (ECC). The x-axis represents genomic position and displays genes within 500kb up- and downstream of the most significantly associated variant for the locus. Y-axis represents the negative logarithm of the association p-value. The blue peaks represent recombination rates at corresponding genomic positions. A. rs76823412; p-value 1.54 × 10^{-6}, B. rs74470773; p-value 4.46 × 10^{-6}, C. rs1044956; p-value 5.35 × 10^{-6}, D. rs9889096; p-value 5.59 × 10^{-6}, E. rs563135; p-value 5.86 × 10^{-6}, F. rs7325099; 7.28 × 10^{-6}, G. rs8091366; p-value 7.60 × 10^{-6}**.**

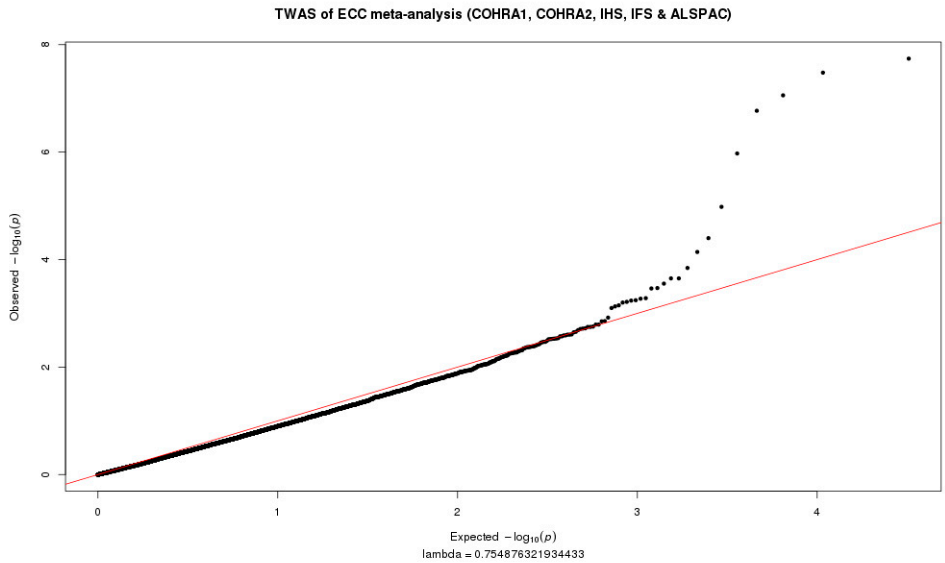

Figure S4. A qq plot of transcriptome-wide association study (TWAS) results of meta-analysis of early childhood caries (ECC) genome-wide association studies (GWASs). The negative logarithms of observed p-values of the GWAS (y-axis) are plotted against the expected p-values from the null hypothesis (x-axis). The inflation factor λ is 0.75.
